# 5,5-Dialkylluciferins are thermal stable substrates for bioluminescence-based detection systems

**DOI:** 10.1371/journal.pone.0243747

**Published:** 2020-12-14

**Authors:** Ce Shi, Michael P. Killoran, Mary P. Hall, Paul Otto, Monika G. Wood, Ethan Strauss, Lance P. Encell, Thomas Machleidt, Keith V. Wood, Thomas A. Kirkland

**Affiliations:** 1 Promega Biosciences, Inc., San Luis Obispo, California, United States of America; 2 Promega Corporation, Madison, Wisconsin, United States of America; CIC bioGUNE, SPAIN

## Abstract

Firefly luciferase-based ATP detection assays are frequently used as a sensitive, cost-efficient method for monitoring hygiene in many industrial settings. Solutions of detection reagent, containing a mixture of a substrate and luciferase enzyme that produces photons in the presence of ATP, are relatively unstable and maintain only a limited shelf life even under refrigerated conditions. It is therefore common for the individual performing a hygiene test to manually prepare fresh reagent at the time of monitoring. To simplify sample processing, a liquid detection reagent with improved thermal stability is needed. The engineered firefly luciferase, Ultra-Glo™, fulfills one aspect of this need and has been valuable for hygiene monitoring because of its high resistance to chemical and thermal inactivation. However, solutions containing both Ultra-Glo™ luciferase and its substrate luciferin gradually lose the ability to effectively detect ATP over time. We demonstrate here that dehydroluciferin, a prevalent oxidative breakdown product of luciferin, is a potent inhibitor of Ultra-Glo™ luciferase and that its formation in the detection reagent is responsible for the decreased ability to detect ATP. We subsequently found that dialkylation at the 5-position of luciferin (e.g., 5,5-dimethylluciferin) prevents degradation to dehydroluciferin and improves substrate thermostability in solution. However, since 5,5-dialkylluciferins are poorly utilized by Ultra-Glo™ luciferase as substrates, we used structural optimization of the luciferin dialkyl modification and protein engineering of Ultra-Glo™ to develop a luciferase/luciferin pair that shows improved total reagent stability in solution at ambient temperature. The results of our studies outline a novel luciferase/luciferin system that could serve as foundations for the next generation of bioluminescence ATP detection assays with desirable reagent stability.

## Introduction

**Scheme 1:**
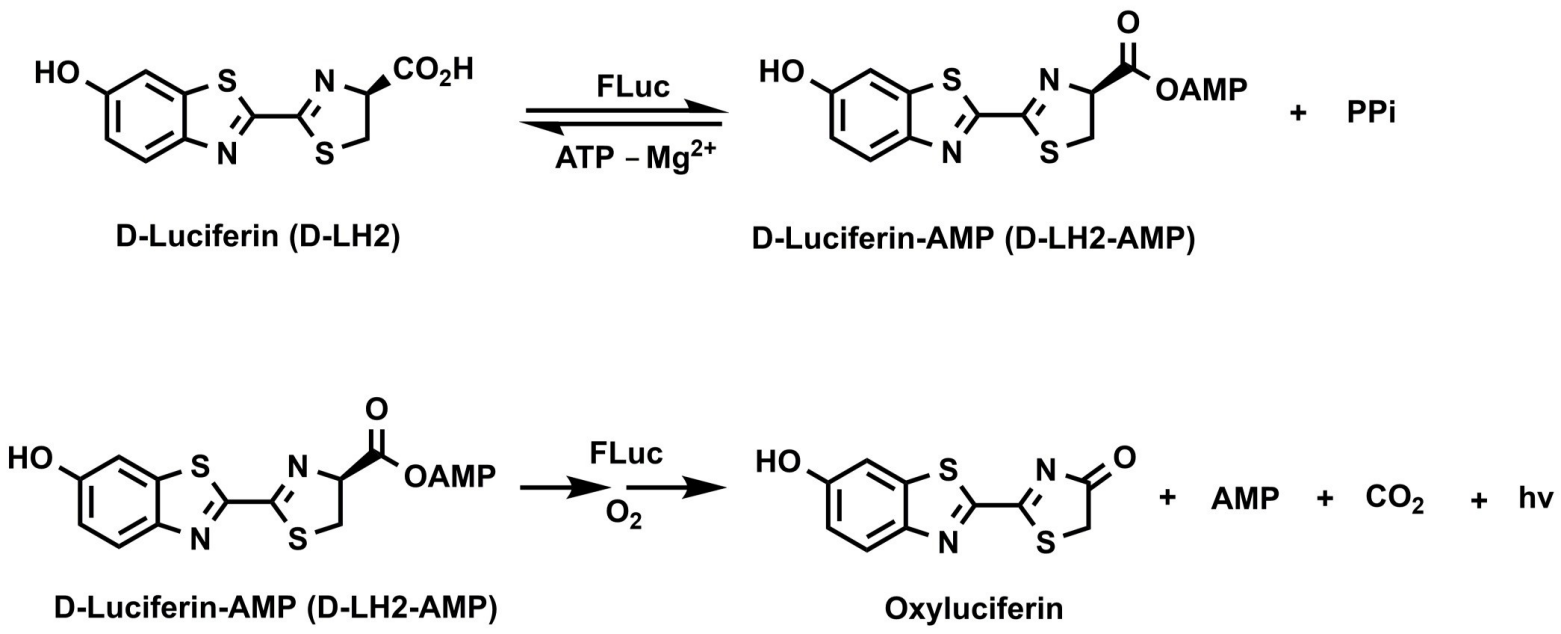
Luciferin chemistry mechanism.

Bioluminescence is well established as a highly sensitive technology for probing biological systems [1–5]. In particular, firefly luciferases and their substrate, D-luciferin (LH2), have been used for a broad range of biological applications in life science research for the past 4 decades (Scheme 1) [6–11]. Unlike fluorescence, which requires an external light source, bioluminescence relies on light-emitting chemical reactions catalyzed by luciferase enzymes. The absence of photon production by the enzyme or substrate themselves results in very low intrinsic background signal enabling a broad range of assay formats and applications with exceptional sensitivity and dynamic range. Recently, the increasing commercial demand for cost-efficient and sensitive detection of chemical or biological contaminants has driven interest in new formats of luciferin-based bioluminescence assays due to their utility, simplicity, and versatility [12–15].

Within commercial settings, ATP quantification assays using bioluminescence have been widely recognized as a fast and cost-effective means for sensitive detection of live microbes from different sample types (e.g., water, crude oil, food) [14, 15]. These assays minimally comprise firefly luciferase and LH2 in an optimized buffer which, upon the addition of ATP present in samples containing live cells, have all the necessary components for rapid generation of a bright, stable, bioluminescence output signal. To date, a major challenge in formulating ATP detection systems for use beyond laboratory settings has been the differing stabilities of enzymes and substrates, resulting in a requirement for separate compositions that are reconstituted and combined at the time of testing. While this strategy helps mitigate stability losses in the system as components with lower stability can be lyophilized or refrigerated, it also increases the complexity of the process and can introduce variability to the assay. In order expand the utility of ATP detection assays to more industrial settings where simplicity and cost-effectiveness are critical, there is a need for both highly thermostable luciferase enzymes and luciferin substrates that can be formulated together in a homogenous, liquid ATP detection system that would enable long-term storage and handling at ambient temperatures without loss of performance.

Progress in improving the thermostability of the firefly ATP detection system up to now has primarily been achieved through formulation studies and protein engineering to increase the stability of the luciferase enzyme component [16–23]. Notably, a thermostable firefly luciferase mutant, trademarked as Ultra-Glo™ [24], has been developed via directed evolution to withstand variations in assay conditions. These include elevated temperature and high concentrations of ionic detergents and reducing agents. Together, these properties enable robust performance under reaction conditions that allow simultaneous cell lysis and bioluminescent detection that could not be achieved with the native luciferase enzyme [25–28].

**Scheme 2:**
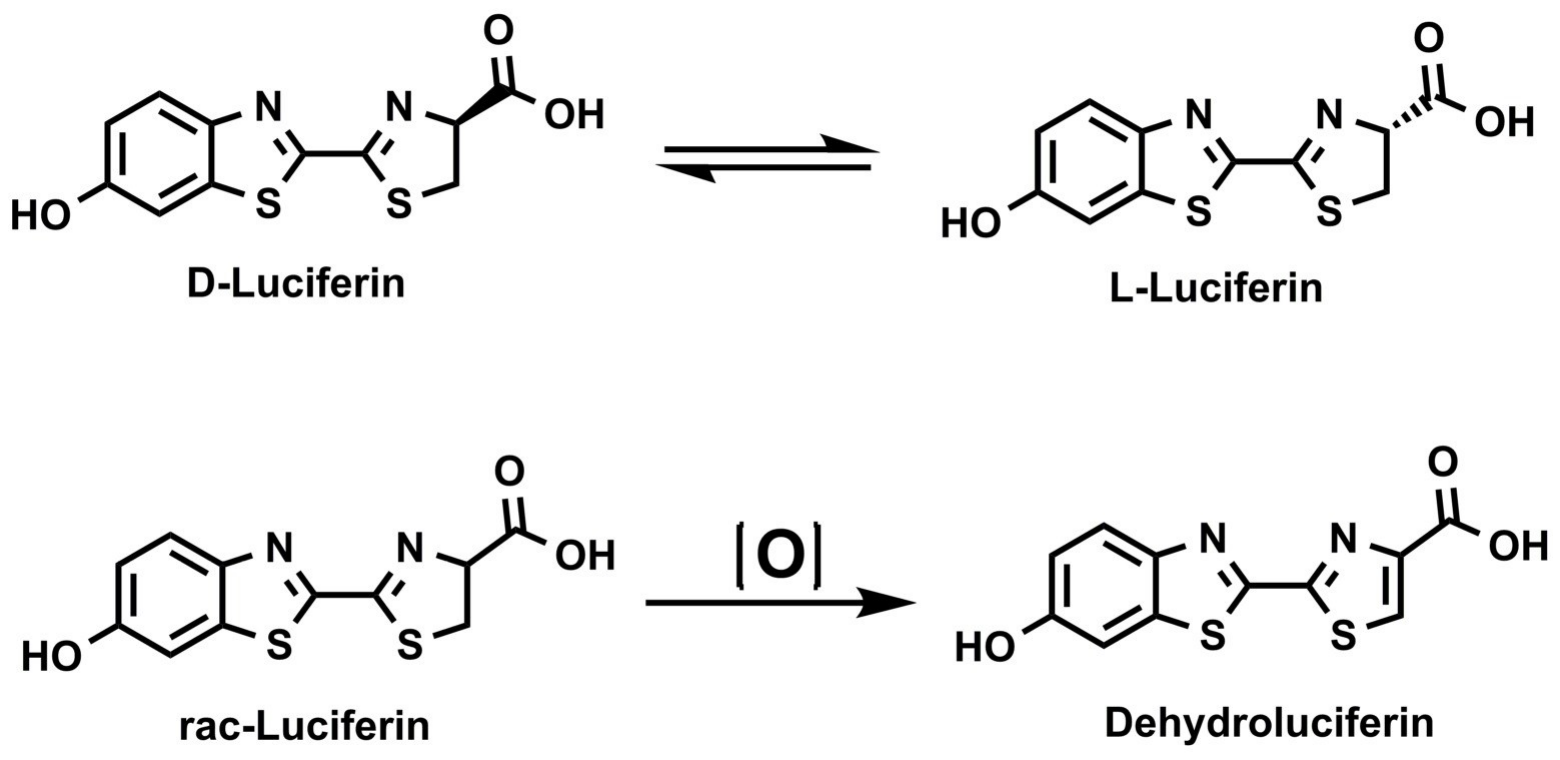
Luciferin breakdown pathway in solution.

The relative stability of luciferin substrates in solution phase, on the other hand, has remained a major challenge to the performance of firefly-based ATP detection systems. This is because ambient storage of LH2 in solution for extended time periods leads to its irreversible decomposition into dehydroluciferin (L), which results in significant loss of ATP detection system performance (Scheme 2) [29]. Mechanistically, dehydroluciferin is a potent inhibitor of firefly luciferases and its accumulation in reagent medium, even at low concentration, compromises assay performance over time [29]. While lyophilization and temperature control strategies have been successful in mitigating formation of dehydroluciferin for laboratory-based applications, they are not able to completely eliminate its accumulation under ambient storage in the homogeneous liquid formats that are desirable for industrial settings.

Here we report a general strategy for improving the solution thermostability of luciferin through substitution of alkyl groups at the 5-position, which prevents oxidative decomposition into dehydroluciferin. By preparing and evaluating a focused library of 5,5-dialkylluciferins, we identified analogs with significantly enhanced thermostability over LH2. Though they were significantly more stable, the analogs are poor substrates for firefly luciferases and require enzymes specifically engineered to utilize them efficiently. To accomplish this, we used directed evolution to engineer variants of Ultra-Glo™ luciferase that could efficiently utilize 5,5-dialkylluciferins as bioluminescence-generating substrates. The results of our studies establish a novel luciferase/luciferin pair that serves as a foundation for next-generation ATP detection systems with high performance and improved reagent stability for industrial applications.

## Materials and methods

### General materials

Reactions were performed using commercially obtained solvents. Unless otherwise stated, all commercially obtained reagents were used as received. Flash column chromatography was performed using pre-packaged RediSep®Rf columns on a CombiFlash Rf system (Teledyne ISCO Inc.). ^1^H and ^13^C NMR spectra were recorded on a Bruker Avance III HD 400 (at 400 MHz and 100 MHz respectively) and are reported relative to internal DMSO-d6 (^1^H, δ = 2.50), CD_3_OD (^1^H, δ = 3.31) and DMSO-d6 (13C, δ = 39.5), CD_3_OD (13C, δ = 49.0). Data for ^1^H NMR spectra are reported as follows: chemical shift (δ ppm) (multiplicity, coupling constant (Hz), integration). Multiplicity and qualifier abbreviations are as follows: s = singlet, d = doublet, t = triplet, q = quartet, m = multiplet, br = broad, app = apparent. High resolution mass spectra were obtained on AB Sciex TripleTOF 5600+. Preparative HPLC was performed with Waters 2535 Quaternary Gradient Module utilizing XBridge PREP C18 Column 5 μm (30 mm X 250 mm). Analytical HPLC was performed with an Agilent 1100 Series HPLC utilizing Phenomenex Synergi™ 2.5 μm MAX-RP 100 Å columns (4.6 mm x 50 mm). 2-cyano-6-hydroxy-benzothiazole (CBT) derivatives (I) were commercially available from Promega Biosciences. β,β-Disubstituted cysteine analogs (II a-h) were either commercially available (II-a from Alfa Aesar) or synthesized via protocols described previously [30, 31]. Ultra-Glo™ luciferase was obtained from Promega Corp.

### Synthesis of 5,5-dialkyllucierins

General procedure: Hydrochloric salts of β,β-disubstituted cysteine analogs (II, 0.15 mmol, 1.5 equiv) dissolved in H_2_O (1 mL) under N_2_ were neutralized with 1 N NaOH (aq, 0.30 mmol, 300 μL, 3.0 equiv). To a solution of 2-cyano-6-hydroxy-benzothiazole (I, 0.1 mmol, 1.0 equiv) in DMF (2 mL) at RT under N_2_, was added the neutralized solution of β,β-disubstituted cysteine analogs. The solution was then stirred under N_2_ for 30 min. LC-MS indicated complete consumption of benzothiazole starting material (I). Luciferins (III) were obtained via preparative HPLC (mobile phase A: 10 mM NH_4_OAc aqueous solution; mobile phase B: CH_3_CN; gradient condition: 5% B to 95% B over 30 minutes).

#### 2-(6-Hydroxybenzo[d]thiazol-2-yl)-5,5-dimethyl-4,5-dihydrothiazole-4-carboxylic acid (III-a)

The product was isolated as white amorphous solid. ^1^H NMR (400 MHz, DMSO-*d6*) δ 7.90 (dd, *J* = 9.0, 0.8 Hz, 1H), 7.41 (s, 1H), 7.13 (dd, *J* = 9.0, 0.8 Hz, 1H), 4.79 (s, 1H), 1.60 (s, 3H), 1.47 (s, 3H).The data is consistent with reported values [32].

#### 5-(6-Hydroxybenzo[d]thiazol-2-yl)-4-thia-6-azaspiro[2.4]hept-5-ene-7-carboxylic acid (III-b)

The product was isolated as off white amorphous solid. ^1^H NMR (400 MHz, DMSO-*d*_6_) δ 13.06 (br s, 1H), 10.23 (br s, 1H), 7.95 (d, *J* = 9.0 Hz, 1H), 7.46 (s, 1H), 7.06 (d, *J* = 9.0 Hz, 1H), 4.98 (s, 1H), 1.42–0.87 (m, 4H); ^13^C NMR (100 MHz, DMSO-*d*_6_) δ 169.2, 165.8, 157.4, 157.2, 146.2, 137.1, 124.9, 117.2, 106.9, 81.7, 40.2, 40.2, 39.9, 39.7, 39.5, 39.3, 39.1, 38.9, 33.7, 17.1, 8.0; HRMS (ESI+) calc’d for C_13_H_11_N_2_O_3_S_2_^+^ [M+H]^+^ 307.0206, found 307.0209.

#### 6-(6-Hydroxybenzo[d]thiazol-2-yl)-5-thia-7-azaspiro[3.4]oct-6-ene-8-carboxylic acid (III-c)

The product was isolated as off white amorphous solid. ^1^H NMR (400 MHz, DMSO-*d*_6_) δ 10.21 (s, 1H), 7.94 (d, *J* = 9.0 Hz, 1H), 7.44 (d, *J* = 2.4 Hz, 1H), 7.06 (dd, *J* = 9.0, 2.4 Hz, 1H), 5.25 (s, 1H), 2.60–2.30 (m, 4H), 2.05–1.73 (m, 2H); ^13^C NMR (101 MHz, DMSO) δ 169.9, 165.0, 162.8, 157.9, 157.6, 146.7, 137.6, 125.3, 117.6, 107.3, 85.9, 63.1, 36.3, 32.8, 31.3, 17.1; HRMS (ESI+) calc’d for C_14_H_13_N_2_O_3_S_2_^+^ [M+H]^+^ 321.0362, found 321.0366.

#### 2-(6-Hydroxybenzo[d]thiazol-2-yl)-1-thia-3-azaspiro[4.4]non-2-ene-4-carboxylic acid (III-d)

The product was isolated as white amorphous powder. ^1^H NMR (400 MHz, DMSO-*d*_6_) δ 10.21 (s, 1H), 7.92 (dd, *J* = 8.8, 1.5 Hz, 1H), 7.44 (s, 1H), 7.05 (d, *J* = 8.8, 1.5 Hz, 1H), 5.15 (s, 1H), 2.14 (d, *J* = 6.9 Hz, 2H), 1.93 (t, *J* = 6.4 Hz, 2H), 1.85–1.63 (m, 4H); ^13^C NMR (100 MHz, DMSO-d6) δ 170.1, 164.9, 157.76, 157.79, 146.7, 137.6, 125.3, 117.6, 107.3, 83.8, 71.4, 41.3, 36.5, 24.2, 23.4; HRMS (ESI+) calc’d for C_15_H_15_N_2_O_3_S_2_^+^ [M+H]^+^ 335.0519 found 335.0526.

#### 2-(6-Hydroxybenzo[d]thiazol-2-yl)-1-thia-3-azaspiro[4.5]dec-2-ene-4-carboxylic acid (III-e)

The product was isolated as white amorphous powder. ^1^H NMR (400 MHz, DMSO-*d*_6_) δ 10.22 (brs, 1H), 7.93 (d, *J* = 8.9 Hz, 1H), 7.44 (d, *J* = 2.4 Hz, 1H), 7.06 (dd, *J* = 8.9, 2.4 Hz, 1H), 4.92 (s, 1H), 2.10–1.49 (m, 8H), 1.46–1.15 (m, 2H); ^13^C NMR (100 MHz, DMSO-*d*_6_) δ 169.9, 164.0, 157.8 (2C), 146.7, 137.6, 125.3, 117.6, 107.3, 86.3, 68.1, 38.9, 34.4, 25.8, 25.3, 24.9; HRMS (ESI+) calc’d for C_16_H_17_N_2_O_3_S_2_^+^ [M+H]^+^ 349.0675 found 349.0677.

#### 5,5-Diethyl-2-(6-hydroxybenzo[d]thiazol-2-yl)-4,5-dihydrothiazole-4-carboxylic acid (III-f)

The product was isolated as off white amorphous powder. ^1^H NMR (400 MHz, DMSO-*d*_6_) δ 7.94 (d, *J* = 8.9 Hz, 1H), 7.44 (d, *J* = 2.4 Hz, 1H), 7.06 (dd, *J* = 8.9, 2.4 Hz, 1H), 5.04 (s, 1H), 2.09–1.94 (m, 1H), 1.90 (dt, *J* = 14.5, 7.4 Hz, 1H), 1.83–1.70 (m, 2H), 1.05–0.85 (m, 6H). ^13^C NMR (101 MHz, DMSO) δ 170.2, 163.6, 157.9, 157.7, 146.7, 137.6, 125.3, 117.6, 107.3, 83.0, 70.7, 31.2, 29.7, 10.8, 10.6. HRMS (ESI+) calc’d for C_15_H_17_N_2_O_3_S_2_^+^ [M+H]^+^ 337.0675, found 337.0679.

#### 2-(6-Hydroxybenzo[d]thiazol-2-yl)-8-oxa-1-thia-3-azaspiro[4.5]dec-2-ene-4-carboxylic acid (III-g)

The product was isolated as white amorphous powder. ^1^H NMR (400 MHz, DMSO-*d6*) δ 7.90 (d, *J* = 9.1 Hz, 1H), 7.40 (s, 1H), 7.09 (d, *J* = 9.1 Hz, 1H), 4.96 (s, 1H), 4.15–3.85 (m, 2H), 3.62–3.42 (m, 2H), 2.50–2.20 (m, 2H), 2.06–1.92 (m, 2H); ^13^C NMR (100 MHz, DMSO-*d*_6_) δ 171.4, 166.3, 159.1, 158.8, 148.1, 139.2, 125.9, 118.3, 107.4, 87.1, 68.4, 67.3, 65.8, 39.5, 35.9; HRMS (ESI+) calc’d for C_15_H_15_N_2_O_4_S_2_^+^ [M+H]^+^ 351.0468 found 351.0472.

#### 2-(6-Hydroxybenzo[d]thiazol-2-yl)-8-methyl-1-thia-3,8-diazaspiro[4.5]dec-2-ene-4-carboxy-lic acid (III-h)

The product was isolated as off white amorphous powder. ^1^H NMR (400 MHz, DMF-*d*_7_ with 3 drops of 0.1 N NaOH aqueous solution) δ 7.93 (d, *J* = 8.9 Hz, 1H), 7.48 (s, 1H), 7.09 (d, *J* = 8.9 Hz, 1H), 4.79 (s, 1H), 3.05–3.00 (m, 2H), 2.37–2.14 (m, 7H), 2.01–1.95 (m, 2H). ^13^C NMR (100 MHz, DMF-*d*_7_ with 3 drops of 0.1 N NaOH aqueous solution) δ 172.0, 161.8, 159.2, 157.4, 146.8, 137.7, 124.9, 117.3, 106.9, 89.8, 65.5, 54.4, 45.1, 38.7, 34.0, 33.5. HRMS (ESI+) calc’d for C_15_H_15_N_2_O_3_S_2_^+^ [M+H]^+^ 335.0519 found 335.0526.

### HPLC-based thermostability profiling

Representative thermal stability profiling of luciferins: Luciferin stock solutions ([LH2]_final_ = 1.0 mM) in Bright-Glo™ assay buffer, with or without Ultra-Glo™ luciferase ([enzyme]_final_ = 0.1 mg/mL; Promega) were incubated at 37 or 60°C. Aliquots (20 μL) were taken out at various time points, diluted with H_2_O (180 μL), and analyzed by RP-HPLC. The percentages of the components were calculated based on UV absorbance at 325 nm.

### Structural modeling of Ultra-Glo™ luciferase with 5,5-dialkyluciferins

A homology model for Ultra-Glo™ luciferase was created from structural templates of firefly luciferases in closed conformation from *Luciola cruciata* (PDB ID: 2D1S) [33] and *Photinus pyralis* (PDB ID: 4G36, chain-B) [34] using Discovery Studio software version 19.1.0.18287 (Dassault Systèmes Biovia Corp). Luciferin analog **III-a** (D and L forms, trans isomers only) were superimposed in the active site of the models based on the positioning of the high-energy intermediate analogue, 5′-*O*-[*N*-(dehydroluciferyl)-sulfamoyl]adenosine (DLSA), present in 2D1S.

### Plasmid construction

Plasmids for expression of Ultra-Glo™ luciferase and mutants were constructed using one-step Gibson assembly kit (SGI) to introduce genes into pF1A plasmid (Promega), resulting in the addition of a C-terminal 8xHis tag to the expressed protein. Constructs were transformed into *Escherichia coli* (*E*. *coli*) KRX chemical competent cells using manufacturers protocols (Promega). Following transformation, individual colony isolates were picked for purification of plasmid DNA using a Wizard SV Miniprep Kit (Promega) and the identity of the cloning region verified by DNA sequencing. All oligonucleotides were purchased from IDT.

### Mutagenesis and library preparation

Individual point mutations were introduced into the Ultra-Glo™ luciferase gene on plasmids first by PCR amplification of the gene with primers encoding the mutation(s) at the site of interest. Amplification products were purified directly from the PCR using a ReliaPrep PCR cleanup kit (Promega). The purified amplicon was then used as a megaprimer to introduce the mutation into the desired plasmid using PCR with Phusion DNA Polymerase (NEB) by combining 20 ng megaprimer with 10 ng unmodified pF1A:Ultra-Glo™. Megaprimer PCR products were used to directly transform *E*. *coli* KRX competent cells, following by plasmid isolation and DNA sequence verification of the target gene. Site-saturation libraries were constructed similar to point mutations except that codons at the mutated site were generated using primers encoded a randomized NNK codon.

Error-prone PCR libraries were created using the Diversify PCR Random Mutagenesis Kit (TaKaRa), following the manufacturer protocol. Randomly mutated PCR amplicons were purified and subcloned into pF1A using Gibson assembly and transformed into *E*. *coli* KRX competent cells. At least ten colonies for each library were selected for DNA sequencing to determine mutation frequency and confirm successful cloning. Libraries with 1–6 mutations/gene were selected for directed evolution screening.

### Directed evolution screening of Ultra-Glo™ luciferase mutants in bacterial lysates

Automated picking of at least 5,000 *E*. *coli* colonies containing individual Ultra-Glo™ luciferase variants per round of screening was performed using a QPix 400 microbial colony picker (Molecular Devices) and used to inoculate LB media plus antibiotic in 96-well plates. Plates were incubated for 16 hours with 400 rpm shaking at 37 ⁰C to allow for outgrowth of the *E*. *coli* culture. A 1:20 dilution of overnight cultures was performed into autoinduction media (LB plus antibiotics, 0.15% Glucose, and 0.1% Rhamnose) in 96-well plates and incubated with 400 rpm shaking at 37 ⁰C for 16 hours to induce protein expression. Cell lysis and luminescence assays were performed in single reactions by mixing induced cultures 1:1 with Detection Reagent Buffer (Promega) containing 1 mM luciferin substrate and 1 mM ATP in a 100 μL total volume. Reactions were incubated for 3 minutes and luminescence measured using a Clariostar plate reader (BMG Biotech). Screening for enzyme variants that improved thermostability was performed by first lysing cells in Detection Reagent Buffer and incubating at 50 ⁰C for 10 min prior to the addition of 1 mM substrate and 1 mM ATP and subsequently measuring the remaining luminescence activity in the culture after 3 minutes. The library size of unique amino acid sequences resulting from introduction of single point mutations in Ultra-Glo™ by error-prone PCR, assuming retention of the start codon and absence of premature stop codons, is 3,184 mutations. By screening at least 5,000 individual clones during each round of directed evolution screening we ensured a minimum of 1.5-fold coverage of all single point mutations in the library. Depending on the selection criteria, this screening coverage typically resulted in 1–2% of clones being identified as hits which were then retested for confirmation and moved into more detailed biochemical characterization experiments as purified proteins.

### Protein purification

Ultra-Glo™ luciferase mutants were purified from 1.5 ml LB autoinduction cultures of *E*. *coli* KRX cells after overnight incubation at 37 ⁰C and 250 rpm shaking. Cells were lysed and His-tagged protein was purified away from cell debris using HisLink Protein Purification Kit (Promega) following the manufacturers protocol. Eluted protein was dialyzed at 4 ⁰C for 16 hours against Storage Buffer (50 mM Hepes pH 7.5, 200 mM NaCl, 1 mM EDTA) and stored at -20 ⁰C.

### K_m_ and V_max_ determination for luciferin substrates

Purified enzyme was diluted to a concentration of 5 μg/ml in Bright-Glo^TM^ buffer (Promega) containing 1 mM ATP and combined in individual reactions with a 10-fold dilution series of substrate ranging from 1pM– 1 mM. Reactions were incubated for 3 minutes at 25 ⁰C prior to measuring luminescence on a GloMax Multi+ Plate Reader. K_m_ and V_max_ were determined using Michaelis-Menten kinetics analysis of activity curves using GraphPad Prism software version 8.4.0.

### Luciferin competition assay

Purified Ultra-Glo™ enzyme was diluted to a concentration of 0.01 mg/ml in 1xTBS + 1% PRIONEX. Separately, a reaction master mix containing 200 nM LH2 + 1 mM ATP was prepared in Bright-Glo^TM^ buffer. The diluted enzyme and reaction master mix were mixed 1:1 in a final volume of 100 μL. Reactions were incubated for 3 minutes at 25 ⁰C prior to measuring luminescence on a GloMax Multi+ Plate Reader. Following initial bioluminescence measurement, 5 μL of 20 mM 5,5-dialkylluciferin in DMSO was spiked into the reactions for a final concentration of 1 mM. Reactions were again incubated at 25 ⁰C for 3 minutes followed by luminescence measurement to determine loss in signal relative to uninhibited samples.

### K_m_ and V_max_ determination for ATP

Purified enzyme was diluted to a concentration of 100 μg/ml in water and combined 1:1 with 100 μM substrate in Buffer A (100 mM MES pH 6.5, 5 mM MgCl2, 0.2% Tergitol, 0.002% Sodium Azide) in a total reaction volume of 100 μl. The mixture was incubated for 60 min at 25 ⁰C to eliminate any background luminescence. Reactions were initiated in individual reactions by spiking in a 50 μl volume of serially-diluted ATP ranging from 1 fM– 1 mM and reading luminescence continuously every 3 seconds for 2 minutes on a GloMax Discover Plate Reader (Promega). The peak luminescence value of each reaction across the reading time was used to calculate the K_m_ and V_max_ for ATP using Michaelis-Menten kinetics analysis of activity curves in GraphPad Prism software version 8.4.0.

### Luminescence-based thermostability profiling

Thermal stability of proteins was determined using Differential Scanning Fluorimetry (DSF) by combining purified protein diluted to a concentration of 0.3 mg/ml in Storage Buffer and adding a final concentration of 5X Sypro Orange dye (Molecular Probes) in a 30 μL reaction volume. Reactions were transferred to Stratagene Mx3000p thermal cycler in a 96-well PCR plate and incubated at 30 ⁰C for 2 minutes prior to heating at a rate of 1°C/min to a final temperature of 100 ⁰C. Fluorescence measurements were taken at one-minute intervals using FAM excitation and ROX emission filters throughout the heating protocol. The transition melting temperature (T_m_) of proteins were calculated from the peak of the first derivative of the fluorescence change over time.

### Luminescence spectra measurements

Assays for measuring luminescence emission spectra were prepared by combining purified Ultra-Glo™ luciferase or mutants at a concentration of 5 μg/ml with 1 mM luciferin substrate and 1 mM ATP in Bright-Glo^TM^ buffer. The emission wavelength spectra of reactions were measured in white polypropylene 96-well plates at 25°C using a Tecan M1000Pro with 10 nm bandwidth, 90 gain, and 2 nm step settings.

## Results and discussion

### Solution thermostability of luciferin is improved with modification at 5-positions

**Fig 1:**
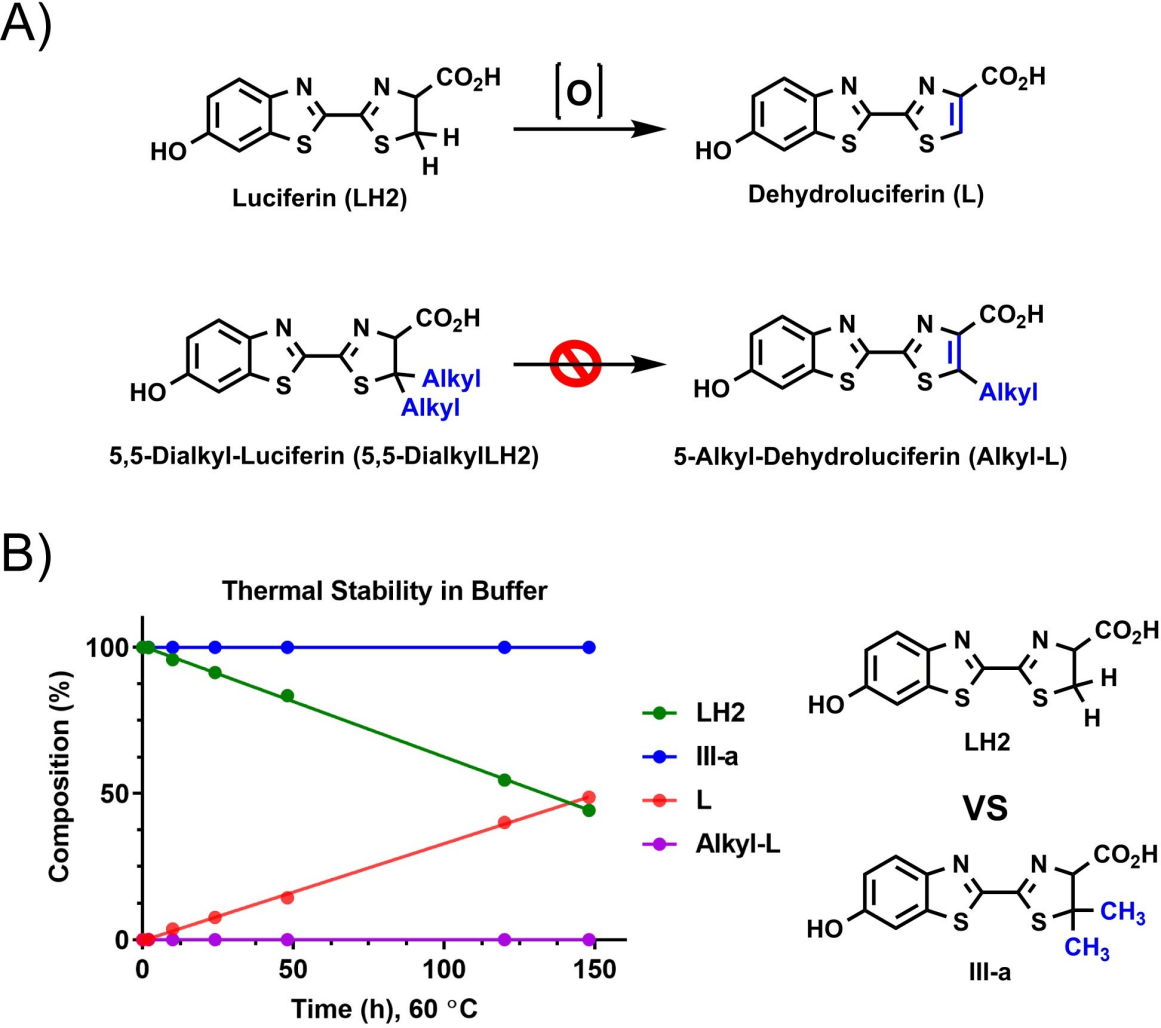
Chemical modification to improve luciferin stability in solution. (A) Hypothesis: 5,5-dialkylluciferins are thermally stable due to their inability to break down into dehydroluciferins. (B) Stability studies confirm that 5,5-dialkylluciferins are thermally stable. 5,5-dimethylluciferin (**III-a**) was compared to unmodified LH2 in an accelerated thermostability study where both compounds (7 mM) were incubated in Bright-Glo^™^ assay buffer at 60°C for 150 hours.

**Scheme 3:**
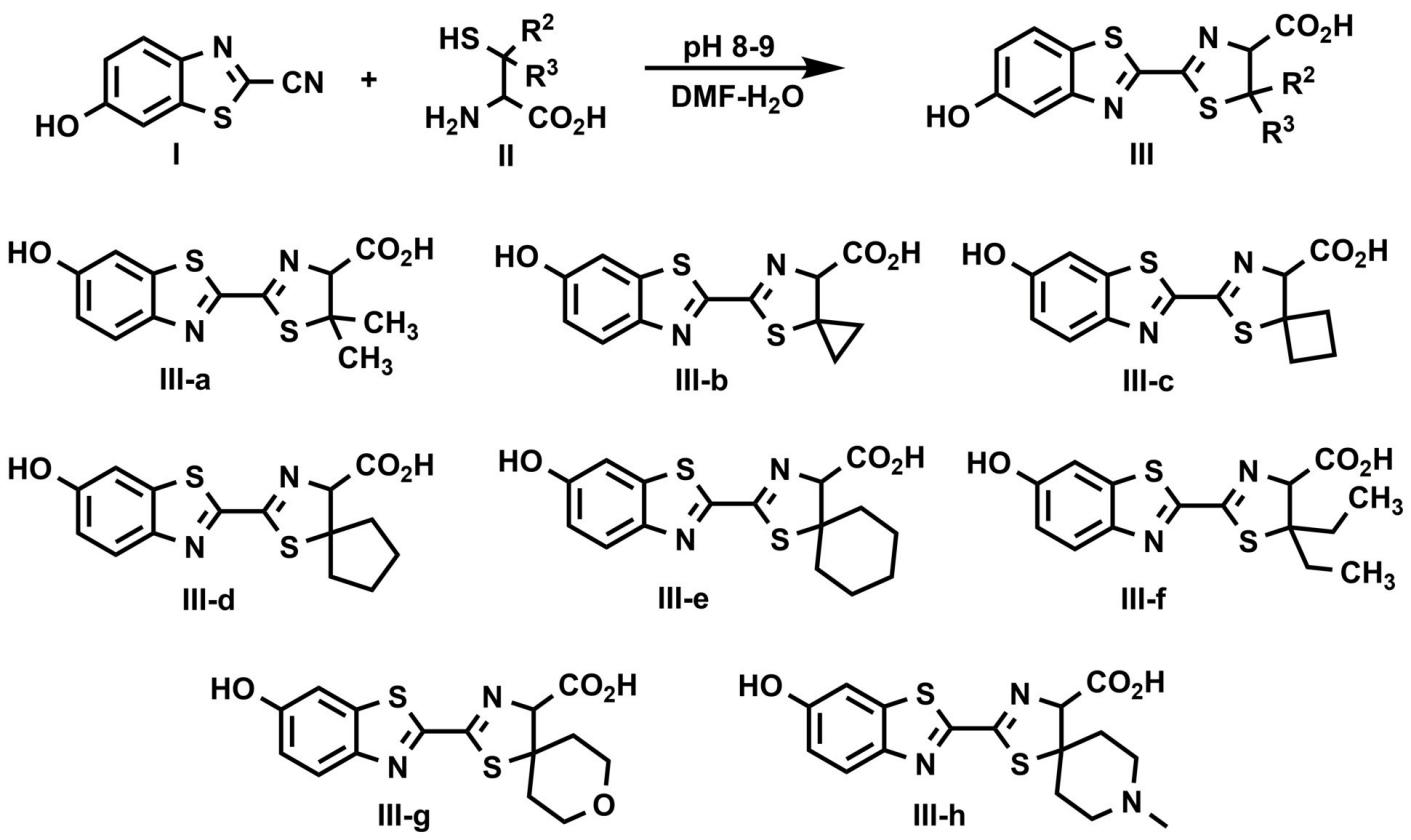
Synthesis of 5,5-Dialkylluciferins. General procedure: Hydrochloric salts of β,β-disubstituted cysteine analogs (II, 0.15 mmol, 1.5 equiv) dissolved in H_2_O (1 mL) under N_2_ were neutralized with 1 N NaOH (aq, 0.30 mmol, 300 μL, 3.0 equiv). To a solution of 2-cyano-6-hydroxy-benzothiazole (I, 0.1 mmol, 1.0 equiv) in DMF (2 mL) at RT under N_2_, was added the neutralized solution of β,β-disubstituted cysteine analogs. The solution was then stirred under N_2_ for 30 min. LC-MS indicated complete consumption of benzothiazole starting material (I). Luciferins (III) were obtained via preparative HPLC (mobile phase A: 10 mM NH_4_OAc aqueous solution; mobile phase B: CH_3_CN; gradient condition: 5% B to 95% B over 30 minutes).

With the goal of improving ATP detection reagent stability in a liquid format at ambient temperature, we decided to explore structure optimization of luciferin (LH2) to determine its impact on formation of dehydroluciferin (L), a major contributing factor to reagent instability. It was hypothesized that replacement of H atoms at 5 positions in the luciferin structure with alkyl substituents could potentially block the oxidation of the thiazoline ring, preventing inhibitory dehydroluciferin accumulation, thereby improving overall assay stability and performance (Fig 1A). To test the hypothesis, 5,5-dimethylluciferin (Scheme 3, **III-a**), an extensively studied luciferin analog [35, 36], was compared to unmodified LH2 in an accelerated thermostability study where both compounds were incubated in solution at 60°C for 150 hours. Remarkably, we observed no degradation for **III-a** over the course of the study, whereas >50% of LH2 decomposed into dehydroluciferin under identical conditions (Fig 1B). Given that the presence of only 3% dehydroluciferin is sufficient to reduce assay performance by 20% (S1 Fig in S1 File), its complete elimination would be predicted to have a significant impact on liquid detection reagent stability. This confirmed our hypothesis that modification of luciferin could improve its thermostability and suggested a general mechanism where two alkyl substituents at the 5 position, are capable of blocking degradation. Therefore, we synthesized and tested other 5,5-dialkylluciferins to identify candidates that are both thermostable and might be suitable as bioluminescent substrates for development into improved liquid detection reagents.

### Synthesis of 5,5-dialkylluciferins

Encouraged by the improved thermostability of 5,5-dimethylluciferin (**III-a**), we set out to explore the 5 position of LH2. A focused set of 5,5-dialkylluciferin analogs were prepared (**Scheme 3**) to explore tolerance of steric hindrance and heteroatom substitution. In general, racemic β,β-dialkyl-cysteines (**II-a-h**) were first synthesized via reported procedures to pre-install the desired alkyl substitutions [30, 31]. Subsequently, racemic 5,5-dialkylluciferins (**III-a-h**) were synthesized via condensation between 6-hydroxy-2-cyanobenzothiazole (**I**) and racemic β,β-dialkyl-cysteines (**II-a-h**) following standard luciferin synthesis procedures (**Scheme 3**). The synthetic process provided convenient access to 5,5-dialkylluciferin analogs with focused structural diversity, allowing us to systematically interrogate analog thermostability, enzyme-substrate interactions, and bioluminescence performance.

### Biochemical evaluation and HPLC-based stability profiling of 5,5-dialkylluciferins

**Fig 2:**
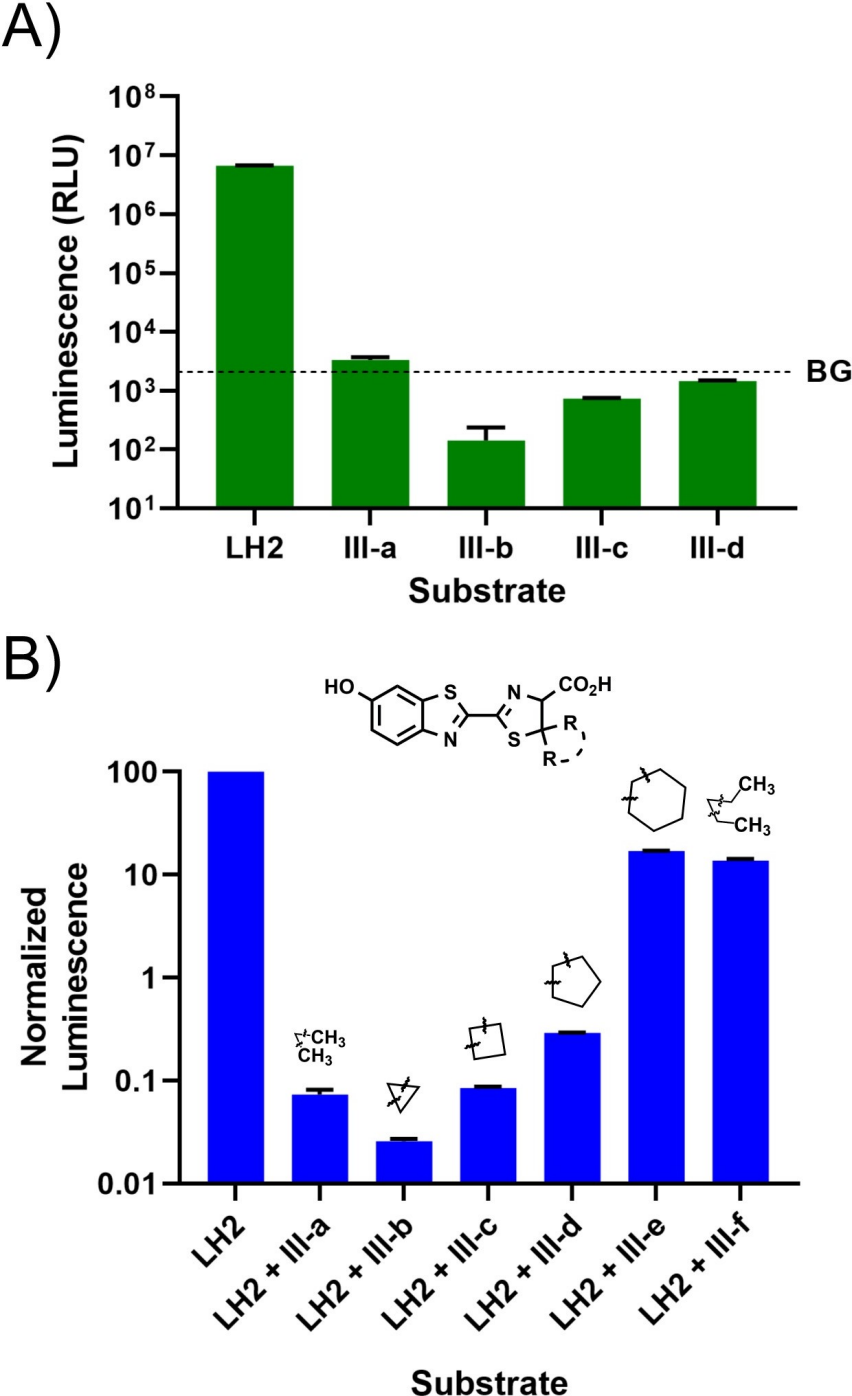
Biochemical evaluation of 5,5-dialkylluciferins. (A) Maximum luminescence of Ultra-Glo™ luciferase with different 5,5-dialkylluciferin substrates. A dotted line indicating background luminescence from a no compound control lacking substrate in Bright-Glo^TM^ buffer provides a baseline comparison to show the reduction in luminescence from reactions containing 5,5-dialkylluciferins. (B) Inhibition of the Ultra-Glo™:LH2 luminescence reaction with 5,5-dialkylluciferins. Reactions containing Ultra-Glo™ luciferase, 1 mM ATP, and 100 μM LH2 were spiked with 1 mM of indicated analog. A normalization factor that converted the raw luminescence value of Ultra-Glo™:LH2 reaction to a value of 100 was applied to all samples in the experiment to show the significant reduction in luminescence of the system when 5,5-dialkyl luciferins were spiked in. Experiments were performed in duplicate and plotted as the average with error bars representing one standard deviation from the mean.

With these novel luciferin analogs in hand, we proceeded to perform biochemical testing to identify lead analogs using a firefly luciferase mutant, Ultra-Glo™, chosen because of its high thermostability and tolerance to chemical additives that are commonly found in homogenous liquid detection reagents. We were careful to identify reaction conditions that would establish a consistent baseline of background luminescence in our assays, since small amounts of activity from the 5,5-dialkylluciferins could easily be hidden due to fluctuations caused by buffer conditions, reagent concentrations, and reaction formulation. Under the conditions tested; however, none of the luciferin analogs showed significant luminescence above background levels (Fig 2A). This result is consistent with another report that **III-a** is not a substrate for firefly luciferase [35, 36].

Although the luciferin analogs did not appear to be substrates for Ultra-Glo™ luciferase, during our testing we noticed that the established background luminescence signal of several reactions in Bright-Glo^TM^ buffer was suppressed in the presence of luciferin analogs such as **III-b** and **III-c**. Given that the background signal of our reactions required the presence of Ultra-Glo™ luciferase, we were intrigued by the observation that the 5,5-dialkylluciferins could inhibit the enzyme-dependent background luminescence of our research enzyme preparations. This suggested that the analogs could interact with the enzyme and that a relative measurement of their inhibition could help us evaluate substrate-enzyme interactions and subsequent potential as substrates for catalysis. We also drew support of their potential as substrates from previous report of 5,5-dialkylluciferin-AMP intermediates being substrates for *Photinus pyralis* and Click Beetle Green luciferases [35].

To gain more insights into substrate-enzyme interactions with this strategy, a bioluminescence-based luciferin competition assay was designed to indirectly assess binding of the luciferin analogs in the active site. We reasoned that analogs that could efficiently bind to the active site would be more successful in competing with LH2 and result in reduced light output in the assay. Indeed, we observed that the relative light output of Ultra-Glo™ luciferase (5 ug/mL) with 100 nM LH2 was reduced in the presence of 1 mM of analogs **III-a-f** (Fig 2B). There was also an inverse correlation between the size of the substituents at 5 positions and the percent of light output inhibition, indicating smaller substituents are better accommodated in the active site. In particular, we noticed analogs **III-a** and **III-b** showed significant inhibition, close to the background level of the assay, suggesting they had the strongest interactions with the enzyme. Based on this result, **III-a** and **III-b** were chosen as lead analogs for further thermostability characterization and subsequent evaluation as enzyme substrates.

**Fig 3:**
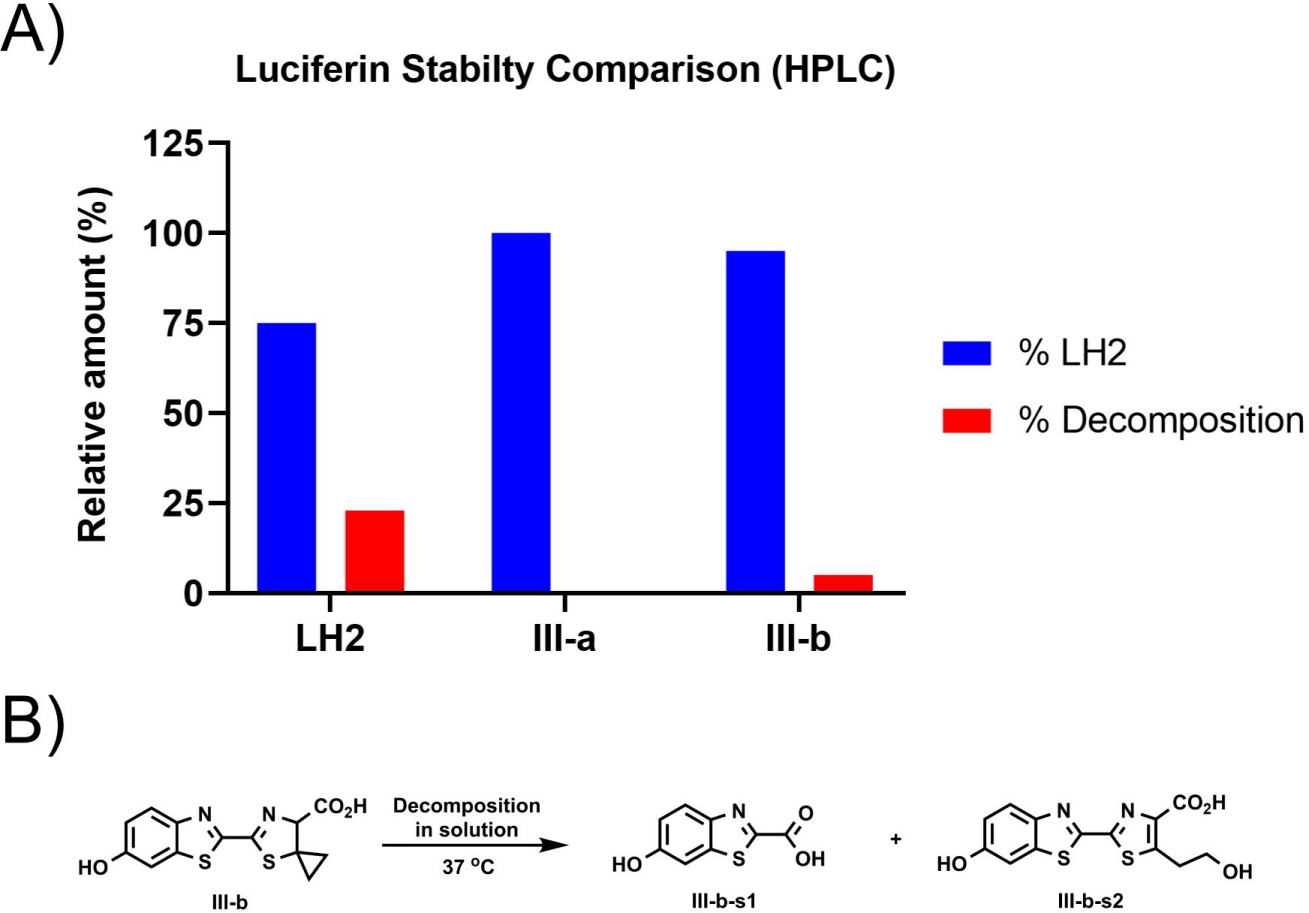
HPLC-based luciferin stability profiles. (A) HPLC-based quantification of luciferin decomposition at 325 nm. 1 mM luciferins (LH2, **III-a** or **III-b**) were reconstituted in Bright-Glo™ buffer and incubated at 37 ^o^C. Aliquots were taken at day 60, percentage of decomposition were quantified based on absorbance at 325 nm. Blue bar represents percentage of luciferins, and red bar represents percentage of decomposition. At day 60, 25% of LH2 were degraded into dehydroluciferin, while **III-b** showed no signs of decomposition. Less than 5% of decomposition were observed for **III-b**. (B) Identification of decomposition products of **III-b**. **III-b-s1** and **III-b-s2** were identified as key decomposition products, the details of enrichment and identification were illustrated in S2 File.

HPLC-based thermostability profiling was conducted to confirm stability of **III-a** and **III-b** in relevant assay buffer and investigate the decomposition products. Luciferin analogs **III-a** and **III-b** were reconstituted at 1 mM in Bright-Glo^TM^ buffer and incubated at 37 ⁰C; taking aliquots at various time points for analysis by RP-HPLC. Over the 2-month period of testing, 23% decomposition of LH2 to dehydroluciferin was observed, whereas no measurable decomposition could be measured for luciferin **III-a** (Fig 3A). Some decomposition (5%) was observed for luciferin **III-b** bearing a spirocyclopropyl at the 5 position. We further investigated the decomposition of **III-b** to evaluate the impact of its individual products on bioluminescence assay performance. Decomposition products of **III-b** were enriched using a modified reaction condition (S2 Fig in S1 File), and the identities were confirmed by ^1^H NMR and HRMS (in S2 File). Decomposition product **III-b-s1** (Fig 3B) is likely to be formed via hydrolysis of the thiazoline ring due to increased ring strain induced by the spirocyclopropane. Decomposition product **III-b-s2** (Fig 3B) is a dehydroluciferin-like analog, which is possibly formed via an oxidative radical ring-opening process involving oxygen. Testing of both decomposition products, **III-b-s1** and **III-b-s2,** in biochemical assays showed only weak to moderate inhibition activities against Ultra-Glo™ mutants that make good utilization of **III-b** (S2 Fig in S1 File), indicating that although III-b exhibits some decomposition in solution the products accumulate more slowly and are less inhibitory than those originating from LH2. Nevertheless, the improved thermostability properties of lead analogs **III-a** and **III-b** over LH2 demonstrated their utility as candidates for liquid-based detection reagents and, when taken together with their strong enzyme interaction potential, highlighted the need to identify luciferase variants that could use them as substrates efficiently.

### Improvement of substrate utilization through structure-guided mutagenesis of Ultra-Glo™ luciferase

**Fig 4:**
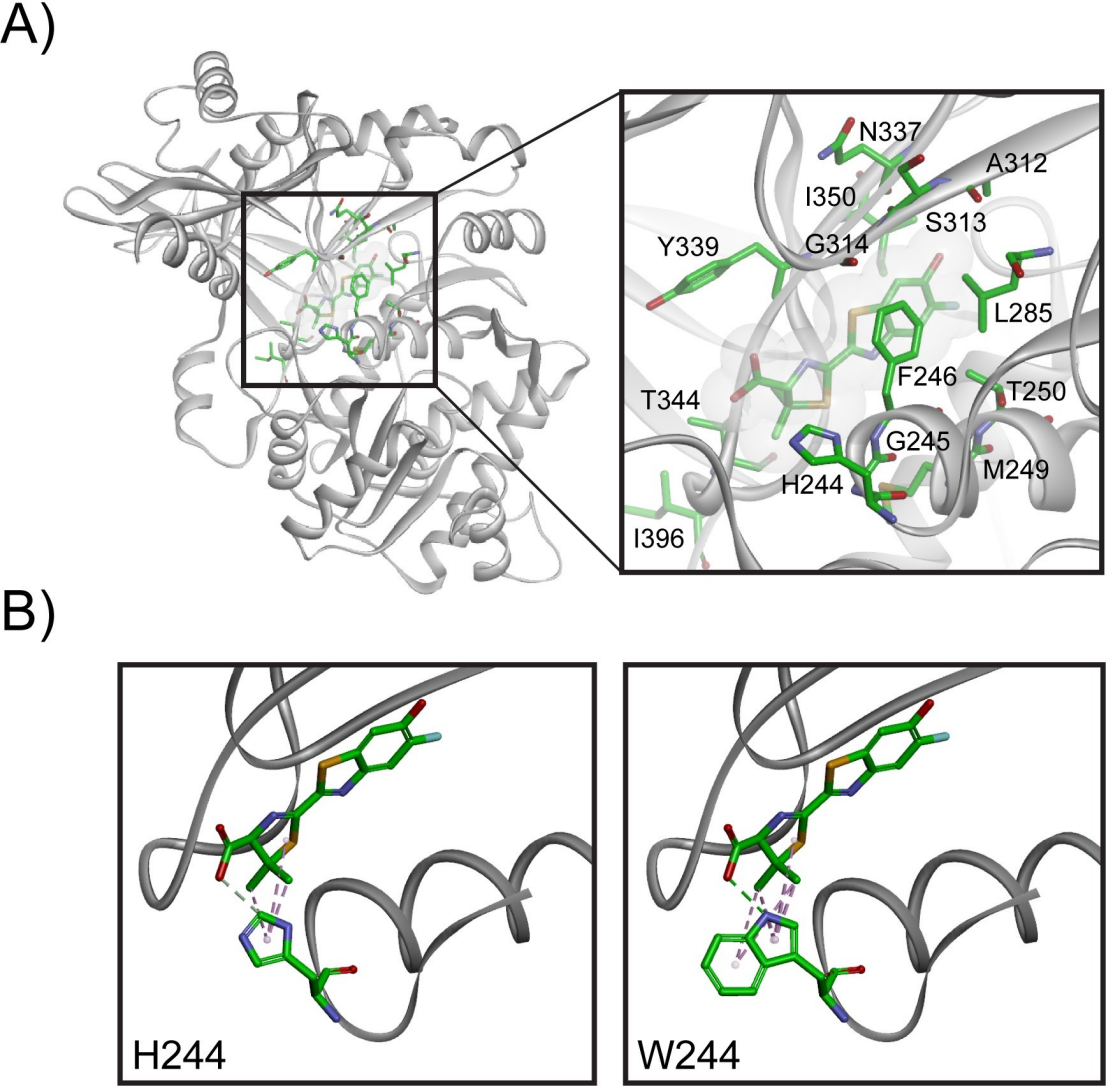
Structure-guided mutagenesis of Ultra-Glo™ identifies a key residue in 5,5-dialkylluciferin utilization. (A) Homology model of Ultra-Glo™ luciferase with 5,5-dimethylluciferin. The expanded view highlights residues in proximity to the active site that were screened by site-saturation mutagenesis. (B) Mutation of H244 to tryptophan had significant improvements in brightness with 5,5-dialkylluciferins and is positioned in close proximity to the dialkyl group of the substrate.

**Fig 5:**
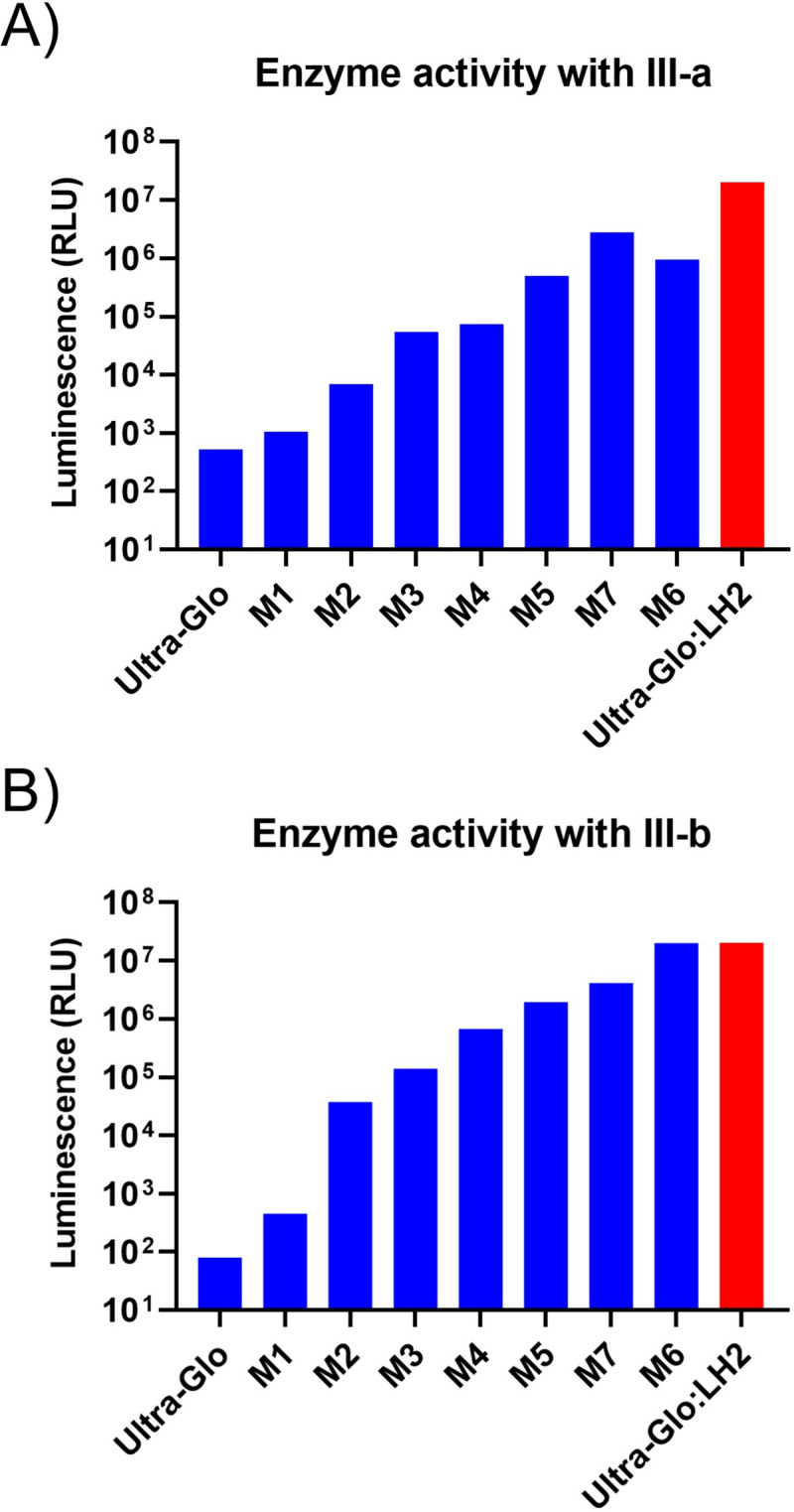
Directed evolution improves the activity of Ultra-Glo™ luciferase with 5,5-dialkylluciferins. Progression of brightness improvements during Ultra-Glo™ luciferase evolution as measured through multiple rounds of high-throughput rational and randomized screening with substrates (A) **III-a** and (B) **III-b**. The activity of the **III-b**:M6 combination closely matched that of LH2:Ultra-Glo™, demonstrating that 5,5-dialkylluciferins can be utilized as highly efficient substrates of bioluminescence reactions.

Although Ultra-Glo™ luciferase does not utilize the 5,5-dialkylluciferins we tested as substrates, our lead 5,5-dialkylluciferins did demonstrate the ability to compete for its native LH2 substrate, suggesting they could bind to the active site in an orientation potentially suitable for catalysis. Therefore, we sought to explore the evolvability of Ultra-Glo™ luciferase toward utilizing them as substrates and enable evaluation of our lead analogs in complete detection reagent formulations in real-time studies. We initially targeted mutagenesis of residues in the active site that would be predicted to influence substrate binding and/or specificity using a three-dimensional homology model of Ultra-Glo™ luciferase based on the structures of the related *Luciola cruciata* (PDB ID: 2D1S) and *Photinus pyralis* (PDB ID:4G36) luciferases in closed-conformation complexes with the high-energy intermediate analogue, 5′-*O*-[*N*-(dehydroluciferyl)-sulfamoyl]adenosine (DLSA) [33, 34]. Using the positioning of the DLSA inhibitor in the native structure as a guide, we superimposed **III-a** in the active site and identified residues in proximity to the substrate that could be determinants in changing the specificity of Ultra-Glo™ for the 5,5-dialkylluciferins (Fig 4A). From this model, 24 amino acid residues within 5 Å of the active site substrate were identified to serve as candidates for mutagenesis. Each of these positions in Ultra-Glo™ was saturated with amino acid substitutions in individual mutant libraries and screened in *E*. *coli* cell lysates for activity against LH2 and **III-b**, followed by secondary screening of hits in smaller batches against **III-a**. A single mutation, H244W, was identified that showed large 17-fold and 88-fold improvements in specific activity with **III-a** and **III-b**, respectively (M2, Fig 5A and 5B). Examining this residue in our homology model showed that this substitution makes additional hydrophobic interactions between 5,5-dialkyl groups and the larger indole sidechain of tryptophan (Fig 4B).

The identification of the H244W mutant provided enough of an improvement in brightness to enable additional screening for Ultra-Glo™ luciferase mutations with **III-b**, while **III-a** was included in secondary assays throughout the process to monitor for general improvements. We next explored changes by incorporating a set of characterized mutations from an in-house repository of Ultra-Glo™ luciferase variants identified during other high-throughput screens of the enzyme that impact substrate utilization. Testing of these additional mutations (M1 and M3, S1 Table in S1 File) with our lead luciferin analogs followed by combinatorial optimization of the best hits resulted in the addition of mutations T344A and I396K to the H244W template which further improved its activity 225-fold with **III-b** over the starting Ultra-Glo™ template (M4, Fig 5B). These mutations are not in close proximity to the active site of our homology model, suggesting they exert their influence on activity through other structural mechanisms. Together, the striking improvements in Ultra-Glo™ from these rationally-guided point mutations provided evidence that the enzyme could be further evolved to efficiently utilize our lead 5,5-dialkyl luciferin analogs and justified a larger-scale, randomized directed evolution effort to identify additional mutations.

### Improvement of substrate utilization through directed evolution of Ultra-Glo™ luciferase mutants

Directed evolution of Ultra-Glo™ luciferase was carried out through successive rounds of introducing diversity through mutagenesis, activity screening, and combinatorial optimization of hits to yield variants with improved brightness and kinetic properties with our lead luciferin analogs. We employed multiple mutagenesis strategies of the full-length gene. We combined random mutagenesis by error-prone PCR of both the existing and a codon-diversified template as well as combinatorial mutagenesis by DNA shuffling. For both strategies, individual variants of Ultra-Glo™ luciferase were overexpressed in *E*. *coli* cultures and tested for activity in cell lysates with the addition of 1 mM ATP and 1 mM of either **III-a** or **III-b**. Variants with 2-fold or greater improvements were selected for secondary screening to further characterize their kinetic profile (K_m_ and V_max_) with each substrate and their DNA sequenced to identify mutations. The brightest combinations of mutations in a single gene were used as the template for the next round of evolution.

Following the initial brightness improvements realized with the triple H244W+T344A+I396K mutant, successive rounds of directed evolution showed continual improvements in brightness (Fig 5), eventually leading to a specific activity level of M6 with **III-b** comparable to that of Ultra-Glo™ and LH_2_ (Fig 5B). Overall, our directed evolution screening was successful in both broadening the substrate specificity of Ultra-Glo™ to include multiple 5,5-dialkylluciferins and identifying variants that could efficiently utilize them for bright luminescence output. Achieving a level of bioluminescence performance close to that of Ultra-Glo™:LH2 is especially notable, considering our lead 5,5-dialkylluciferin analogs were actually inhibitors of our starting Ultra-Glo™ luciferase template prior to evolution.

Two Ultra-Glo™ luciferase mutants, M6 and M7, resulting from our evolution strategy were chosen for more detailed biochemical characterization since they had the most significant brightness improvements with **III-a** and **III-b** while retaining relatively high thermostability. Comparison of V_max_ values between Ultra-Glo™ luciferase and the two mutant enzymes showed that M6 paired with **III-a** and **III-b** had V_max_ values of 2.8 x 10^6^ and 2.7 x 10^7^ RLU, respectively; the latter of which is about 1.5-fold brighter than the specific activity of Ultra-Glo™ with LH_2_ (Table 1). M7, a variant evolved concurrently to improve the enzyme thermostability over M6, retains the preference for utilization of **III-a** and **III-b** although with slightly lower V_max_ values of 2.4 x 10^6^ and 3.5 x 10^6^ for the two substrates (Table 1). Comparison of other kinetic properties for mutants M6 and M7, summarized in Table 1, showed both mutant enzymes exhibit a red shift of ~40 nm in their peak luminescence signal independent of the substrate tested and have higher Km for the 5,5-dialkylluciferins than LH2.

**Table 1:** Kinetic and thermostability parameters of evolved Ultra-Glo™ mutants with III-a and III-b.

Enzyme	Substrate	V_max_ (RLU)	K_m_ (μM)	λ_max_ (nm)	K_m_, ATP (μM)	DSF T_m_ (°C)
Ultra-Glo™	LH2	1.8 x 10^7^	0.67	557	0.46	80
	III-a	-	-	-	-	
	III-b	-	-	-	-	
M6	LH2	1.4 x 10^6^	0.13	597	3.11	70
	III-a	2.8 x 10^6^	5.36	600	0.4	
	III-b	2.7 x 10^7^	14.03	597	1.75	
M7	LH2	5.5 x 10^6^	0.49	597	1.64	75
	III-a	2.4 x 10^6^	7.47	600	1.16	
	III-b	3.5 x 10^6^	1.71	600	6.58	

Since we observed that many of the mutations selected during directed evolution with **III-b** also led to improvement utilization of **III-a** by Ultra-Glo™ mutants, we were curious if the other 5,5-dialkyl luciferins in our original panel would have comparable improvements as well. For unmutated Ultra-Glo™ luciferase, little or no luminescence could be observed across the panel of luciferin analogs we tested (S3 Fig in S1 File). However, the evolved mutants M6 and M7 were able to utilize multiple analogs including **III-a**, **III-b**, **III-c**, and **III-d** (S3 Fig in S1 File). The modifications in this subset are comprised of 5,5-dimethyl and a series that included 5,5-cyclopropyl, 5,5-cyclobutyl, and 5,5-cyclopentyl ring substitutions. These results demonstrate that the improvements to Ultra-Glo™ luciferase through directed evolution with **III-b** partially translated to other 5,5-dialkyl luciferin analogs and highlight the structural plasticity accommodated by the chemical mechanism of bioluminescence in the firefly-based system.

### Thermostability profiling of Ultra-Glo™ mutants

While our evolution program was primarily focused on enhancing the utilization of 5,5-dialkyl luciferins with Ultra-Glo™ luciferase in order to evaluate their potential as substrates for single-liquid ATP detection systems, we also evaluated in parallel the thermostability of our mutant enzymes relative to the highly-stable Ultra-Glo™ parental template. Using differential scanning fluorimetry (DSF), we calculated the transition melting temperature (T_m_) of the purified enzymes as they were exposed to an increasing temperature gradient. We observed that our brightest mutant, M6, had a 10°C reduction in T_m_ relative to Ultra-Glo™ luciferase, indicating that among the combination of amino acid substitutions selected for 5,5-dialkylluciferin utilization included some detrimental to the enzyme’s thermostability (Table 1). Based on DSF data from enzymes that were intermediates in the evolution process, we were able to identify several mutations in M6 that were responsible for the decrease in stability. After reverting several of these mutations to their starting amino acid in Ultra-Glo™ and further directed evolution screening to identify additional stabilizing mutations, we were able to increase the T_m_ to 75 ⁰C in mutant M7, albeit at the expense of its V_max_ with our lead analogs. With its high thermostability and brightness with 5,5-dialkyl luciferins, mutant M7 had an appealing set of properties that would enable initial testing of our highly stable luciferin analogs as components of a homogenous, liquid ATP detection system.

### Evaluating 5,5-dialkylluciferins for use in single-liquid ATP detection systems

The firefly luciferase-based ATP detection system has been widely adopted for monitoring cell health in life science research [37, 38], and as first-line detection of microbial pathogens in industrial settings [39–41]. Currently, single-liquid ATP detection reagents require storage under at least refrigeration conditions (4 ⁰C) to maintain reasonable stability (T_80,_ timing maintaining 80% initial activity, greater than 30 days). Therefore, improvements in the stability of single-liquid ATP detection reagents are highly desired to allow storage at ambient temperature, enabling accurate and reliable detection of ATP in industrial settings in simplified formats without the need for dedicated laboratory equipment or personnel. To evaluate the impact of enzyme and substrate stability in a relevant assay context, we decided to use bioluminescence-based, single-liquid ATP detection system as the testing model to compare the current Ultra-Glo™: LH2 detection system with the newly evolved M7:**III-a** system comprising our most stable components.

We performed three parallel stability studies to compare both systems side by side at 37 ⁰C for 150 days. Within each study, three replicate 1 mL formulations were tested that comprised 1 mM substrate and 0.5 mg/mL enzyme in Detection Reagent Buffer, which allows simultaneous capacity for cell lysis and bioluminescence detection. Throughout the studies, aliquots were taken at indicated time-points and assayed for luminescence by addition of 1 mM ATP. The thermostability parameter, T_80_, was determined by plotting time versus luminescence. Control formulations containing only substrate or enzyme alone were also included, with the held-out component spiked in at the time of activity measurement at each time-point.

**Fig 6:**
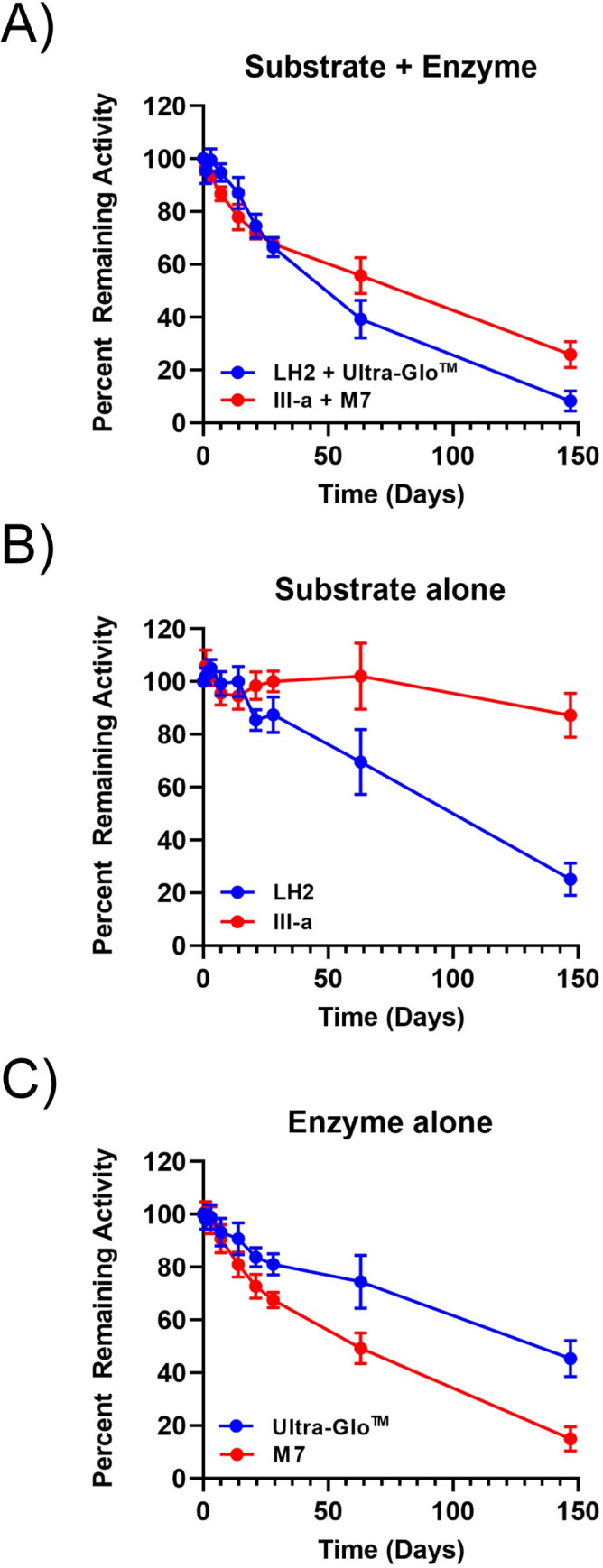
Real-time stability of ATP detection reagents using 5,5-dimethylluciferin. Percent of remaining activity of formulations over 150 days at 37°C in a liquid format comparing the performance of individual components and the complete systems (A) Ultra-Glo™:LH2 and M7:**III-a** combinations, (B) LH2 or **III-a** substrates alone, and (C) Ultra-Glo™ or M7 luciferase enzymes alone. System instability for Ultra-Glo™: LH2 is driven primarily by the decomposition of LH2 into dehydroluciferin, while the 5,5-dimethyl modification in the M7:**III-a** system stabilizes the substrate component. Six replicates were performed for each sample and are plotted as the average at each timepoint with error bars indicated one standard deviation of the mean.

Comparison of real-time stability profiles between the complete single-liquid reagents and stand-alone controls revealed different critical factors for the performance of the Ultra-Glo™:LH_2_ and M7:**III-a** systems. For Ultra-Glo™:LH2, both Ultra-Glo™ and LH_2_ lost ~20% of initial activity based on brightness within 30 days of incubation at 37 ⁰C (Fig 6A). Since 80% of 1 mM LH2 still saturates Ultra-Glo™ luciferase light production, the fact that the complete reagent formulation lost ~40% of initial activity within the same period confirmed that formation of dehydroluciferin has significant impact on complete reagent stability at ambient temperatures. For the M7:**III-a** system, it was noted that **III-a** alone maintained exceptionally high thermostability with very little change to its luminescence profile (Fig 6B), which is consistent with HPLC-based analysis of accelerated stability studies. Although there was similar initial reagent stability for the complete M7:**III-a** system (T_80_ = ~15 d) and the existing Ultra-Glo™:LH_2_ system (T_80_ = ~20 d), there was an ~25% increase in stability for the new system at timepoints beyond 60 days. This improvement in long-term stability for the M7:**III-a** system is likely due to the presence of **III-a**, as its superior stability relative to LH2 in substrate-alone controls became more prominent at these later timepoints in the study. In addition, since the stability trend for the M7:**III-a** was nearly identical to M7 alone, it is clear that the stability of enzyme component is the limiting factor now that the decomposition of the substrate has been eliminated (Fig 6A and 6C). Together, the results of using an ATP detection assay as a model confirmed that the high thermostability of the luciferin analog, **III-a,** translates into improved real-time performance in a homogenous liquid detection format, providing essentially no loss in performance over 5 months of storage at 37°C/98.6°F.

## Conclusion

We have improved thermostability of the standard firefly luciferase substrate, LH2 through chemical modification to create 5,5-dialkylluciferins that reduce or eliminate oxidative decomposition to dehydroluciferin, a potent inhibitor of firefly luciferases and a major limitation for firefly-based ATP detection systems. Despite their initial poor utilization as substrates by Ultra-Glo™ luciferase, 5,5-dialkylluciferins **III-a** and **III-b** were found to display favorable interactions with the enzyme using a luciferin displacement assay. Subsequent direct evolution of Ultra-Glo™ with these analogs yielded a set of luciferase mutants that showed efficient utilization of them as substrates. Notably, the combination of mutant M6 and **III-b** produced a bioluminescence signal comparable in strength to that of Ultra-Glo™ luciferase and LH2.

The importance of discovering a bioluminescent substrate resistant to spontaneous oxidative decomposition here was demonstrated by real-time stability assessment of a homogenous solution containing **III-a** and the luciferase mutant, M7. Throughout the course of a 5-month, real-time stability study we observed no loss in stability or decomposition of **III-a** in solution at 37°C. In a complete formulation, the pairing was ~25% more stable compared to the existing Ultra-Glo™:LH2 system across the length of the study and appeared to only be limited by the eventual loss of stability of the M7 enzyme component. This suggests that further engineering of stability into the luciferase variant to match that of **III-a** would be the next step in developing a homogenous, liquid ATP detection reagent with even higher stability. Our results show that as both individual and combined components, 5,5-dialkylluciferins and thermostable Ultra-Glo™ luciferases form the foundation of a high performance, ATP detection platform for users in industrial or other settings where reagent stability and simple workflow are of critical importance.

More broadly, this novel combination also expands the available modifications of luciferins, providing new tools for potential development of the firefly system toward additional color modulations and pro-luciferin chemistries. Such substrates, with less potential to be oxidized into dehydroluciferin, could potentially improve a wide range of luciferin-based bioluminescence assays. For example, improvements to the luminogenic substrates used in oxidation assays for cytochrome P450 family enzymes could further enable homogenous and high-throughput formats by increasing substrate stability during assay setup and screening [11]. Similarly, bioluminescence assays for reactive oxygen species (ROS) could benefit from more stable, less-oxidatively sensitive 5,5-dialkylluciferins for measuring ROS activity in mitochondria during live-cell based measurements, including those performed in multiplex format with other cell-health detection assays [42]. Together, the improved stability and robustness of both enzyme and substrate components will continue to enable researchers to take advantage of the exceptional sensitivity and flexibility of bioluminescence assays in the future.

## Supporting information

S1 File (DOCX)

S2 File (PDF)
