# Supplementary material for: 5,5-Dialkylluciferins are thermal stable substrates for bioluminescence-based detection systems
Source: PLoS One. 2020 Dec 14;15(12):e0243747. doi: 10.1371/journal.pone.0243747 (PMC7735563; doi:10.1371/journal.pone.0243747)
Supplement: S1 File — (DOCX) [file pone.0243747.s001.docx]

5,5-Dialkylluciferins are thermal stable substrates for bioluminescence-based detection systems

Ce Shi^1¶^, Michael P. Killoran^2¶^*, Mary P. Hall^2^, Paul Otto^2^, Monika G. Wood^2^, Ethan Strauss^2^, Lance P. Encell^2^, Thomas Machleidt^2^, Keith V. Wood^2^, and Thomas A. Kirkland^1^.

Table of Contents

S1 Figure. Dehydroluciferin impact to apparent detection reagent stability................................... 2

S2 Figure. Identification and characterization of decomposition products of III-b...........................3

S3 Figure. Utilization of 5,5-dialkylluciferins by Ultra-Glo™ mutants..........................................4

S1 Table. Mutations present in Ultra-Glo™ variants………………………………………..........5

HPLC-based accelerated thermostability profiling..........................................................................6 Inhibition of luciferase activity by dehydroluciferin………….....………………..……...…......... 6

Synthesis and Identification of III-b-s1 and III-b-s2………………………………………....….....6

Determination of K_i_ of III-b-s1 and III-b-s2……………………………..………………….......…7

References………...………………………...……………………………………………………..8

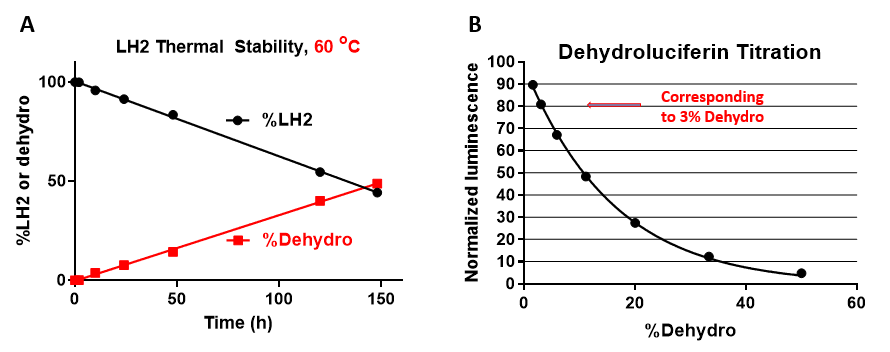


**S1 Figure**. Dehydroluciferin impact to apparent detection reagent stability.


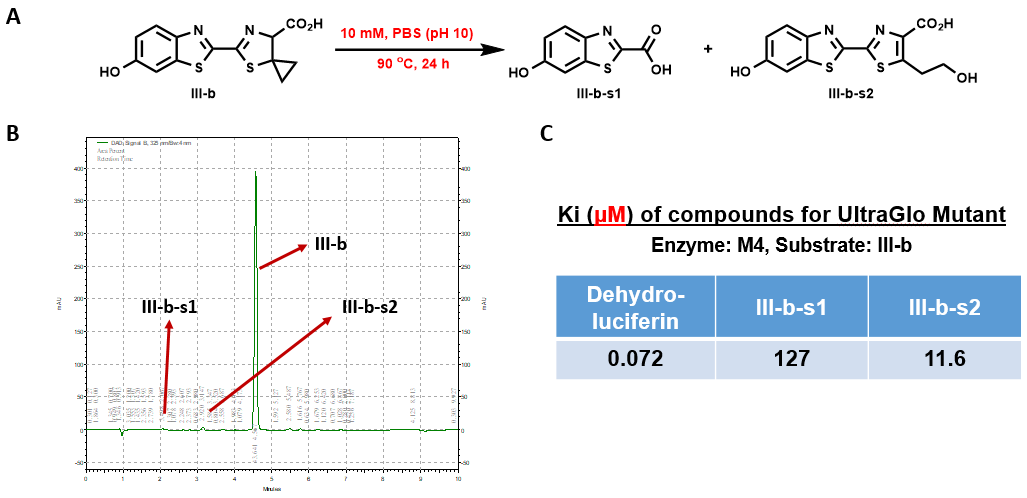


**S2 Figure**. Identification and characterization of decomposition products of III-b.


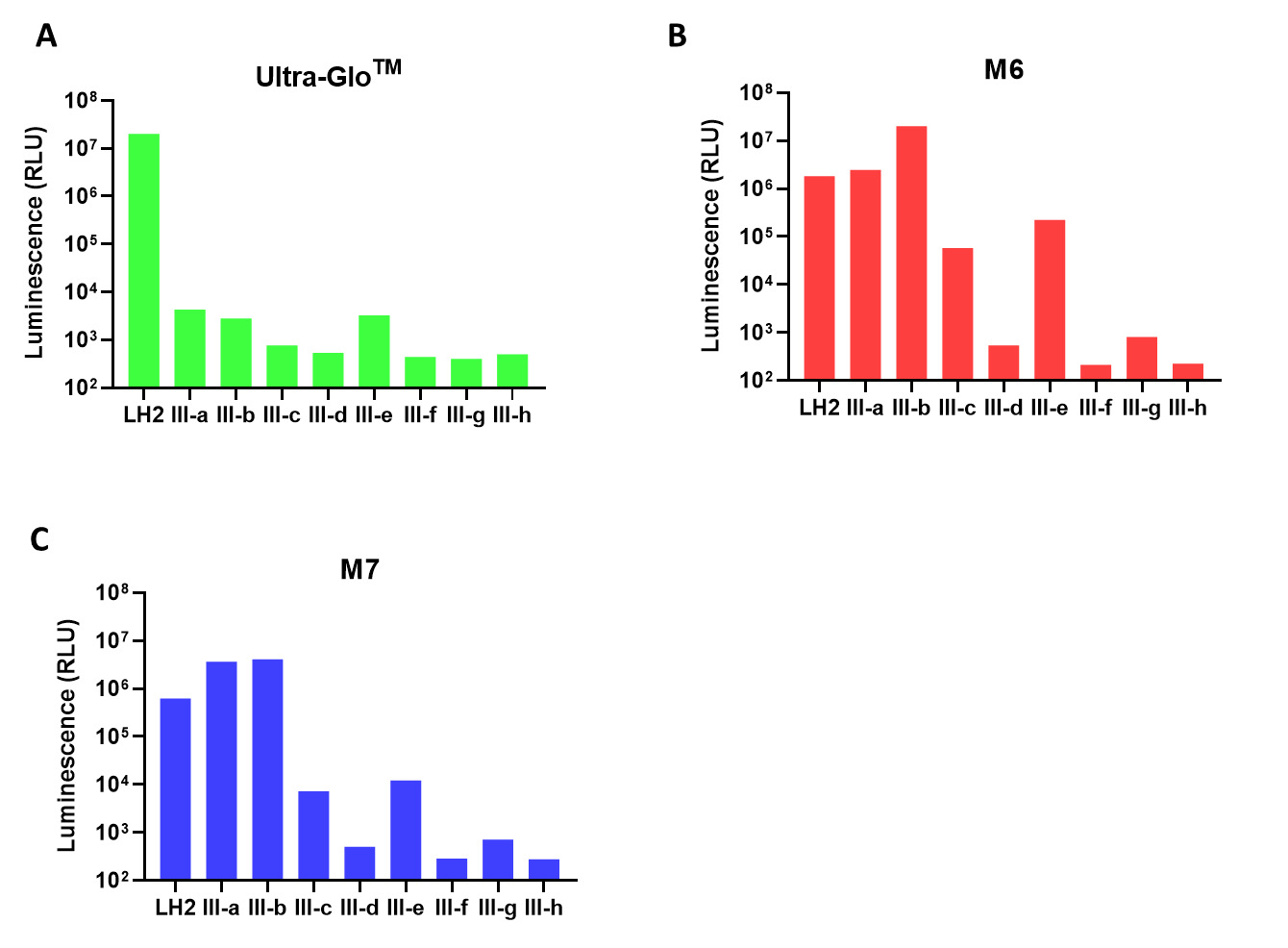


**S3 Figure.** Utilization of 5,5-dialkylluciferins by Ultra-Glo™ mutants.

**S1 Table.** Mutations present in Ultra-Glo™ variants.


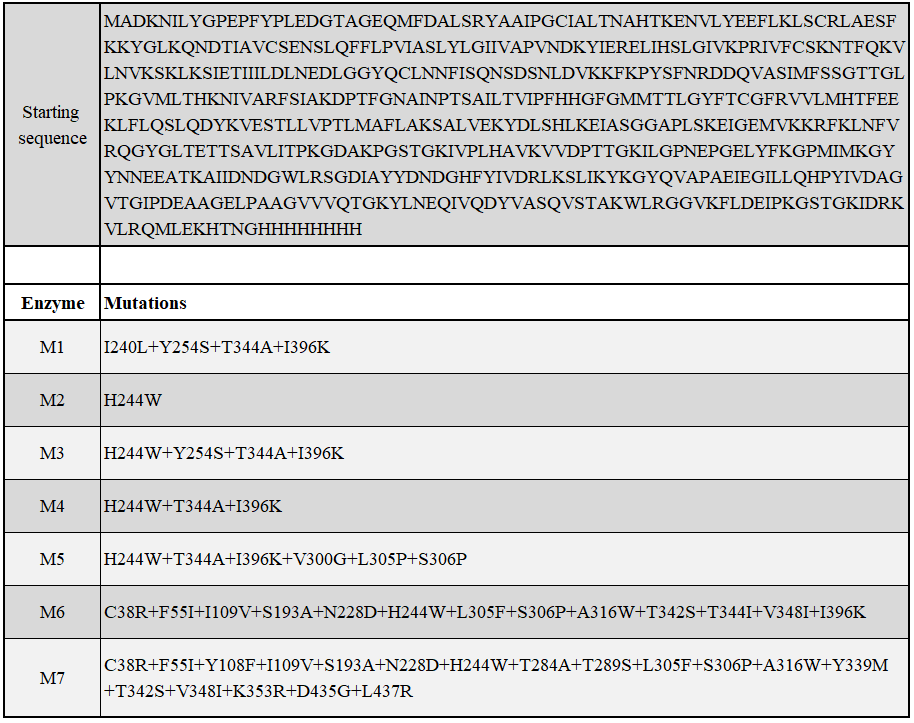


**HPLC-based accelerated thermostability profiling**

Representative thermal stability profiling of luciferins: Luciferin stock solutions (pH= 6.0, [LH2]_final_= 1.0 mM) containing various amounts of detergents, with or without Ultra-Glo™ luciferase ([enzyme]_final_= 0.1 mg/mL; Promega) were incubated at 60 °C. Aliquots (20 μL) were taken out at various time points, diluted with H_2_O (180 μL), and analyzed by RP-HPLC. The percentages of the components were calculated based on UV absorbance at 330 nm. Inhibition of luciferase activity by dehydroluciferin was performed by reconstituting Ultra-Glo™ luciferase (Promega, 0.1 mg/mL) and LH_2_ (0.25 mM) in Detection Reagent buffer +0.1% PRIONEX^®^. 2X serial dilutions of each of the dehydroluciferin samples were prepared using the racemic solutions as a diluent. (500 μL of dehydroluciferin solution added to 500 μL of Ultra-Glo™ + LH_2_). 50 μL of each titration series was then added to 50 μL 0.1 mM ATP.  The samples were incubated for 1 minute and the luminescence was measured on GLOMAX®-Multi+ plate luminometer (n=6).

**Inhibition of luciferase activity by dehydroluciferin**

Ultra-Glo^TM^ luciferase (Promega, 0.1 mg/mL) and LH_2_ (0.25 mM) was reconstituted in in detection reagent buffer +0.1% PRIONEX^®^. 2X serial dilutions of each of the dehydroluciferin samples were prepared using the racemic solutions as a diluent. (500 μL of dehydroluciferin solution added to 500 μL of Ultra-Glo^TM^ + LH_2_). 50 μL of each titration series was then added to 50 μL 0.1 mM ATP.  The samples were incubated for 1 minute and the luminescence was measured on GLOMAX®-Multi+ plate luminometer (n=6).

**Synthesis and Identification of III-b-s1 and III-b-s2**

III-b (6.1 mg, 20 μmol) was reconstituted in PBS buffer (pH 10, 2 mL) to make a 10 mM solution. The solution was incubated at 90 ⁰C for 24 h and used directly for purification. The decomposition products III-b-s1 and III-b-s2 were isolated via preparative HPLC (mobile phase A: 10 mM NH_4_OAc aqueous solution; mobile phase B: CH_3_CN; gradient condition: 5% B to 95% B over 30 minutes).

**6-hydroxy-2-benzothiazolecarboxylic acid (III-b-s1)**. The product was isolated as white amorphous solid. ^1^H NMR (400 MHz, DMF-*d7*) δ 10.20 (br, s, 1H), 8.00 (d, J = 9.0 Hz, 1H), 7.46 (d, J = 2.4 Hz, 1H), 7.10 (dd, J = 9.0, 2.4 Hz, 1H). HRMS (ESI+) calc’d for C_8_H_6_NO_3_S^+^ [M+H]^+^ 196.0062, found 196.0063. The data is consistent with the reported values.^1^

**2-(6-Hydroxybenzo[d]thiazol-2-yl)-5-(2-hydroxyethyl)-4,5-dihydrothiazole-4-carboxylic acid (III-b-s2).** ^1^H NMR (400 MHz, DMF-*d7*) 10.39 (s, 1H), 7.95 (d, *J* = 8.8 Hz, 1H), 7.60 (d, *J* = 2.4 Hz, 1H), 7.16 (dd, *J* = 8.8, 2.4 Hz, 1H), 3.87 (t, *J* = 6.0 Hz, 2H), 3.54 (t, *J* = 6.0 Hz, 2H). HRMS (ESI+) calc’d for C_13_H_11_N_2_O_4_S_2_^+^ [M+H]^+^ 323.0155, found 323.0156.

**Determination of K_i_ for III-b-s1 and III-b-s2**

The inhibition profile of different decomposition products was determined by combining individual reactions containing 5 µg/ml purified enzyme with 1 mM ATP and 50 µM substrate in Bright-Glo^TM^ buffer with a 5-fold serial dilution range of inhibitor from 3.5 nM – 2.5 mM. The K_i_ value of the luminescence data was calculated using GraphPad Prism software version 8.4.0 for competitive enzyme kinetic analysis of inhibition constants.

**References**

1. Borza, I., Bozo, E., Barta-Szalai, G., Kiss, C., Tárkányi, G., Demeter, A., Gáti, T., Háda, V., Kolok, S., Gere, A. and Fodor, L., *J. Med. Chem.* 2007, 50, 5, 901–914.
