# Supplementary figures and images for: 5,5-Dialkylluciferins are thermal stable substrates for bioluminescence-based detection systems
Source: PLoS One. 2020 Dec 14;15(12):e0243747. doi: 10.1371/journal.pone.0243747 (PMC7735563; doi:10.1371/journal.pone.0243747)

7.91  
7.89  
7.41  
7.40  
7.14  
7.14  
7.13  
7.12  
7.12  
7.11  
7.11

1.77  
1.53

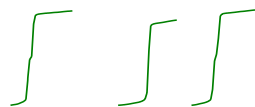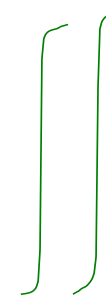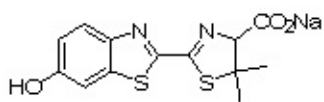

III-a

1.00  
0.90  
1.01

2.86  
3.03

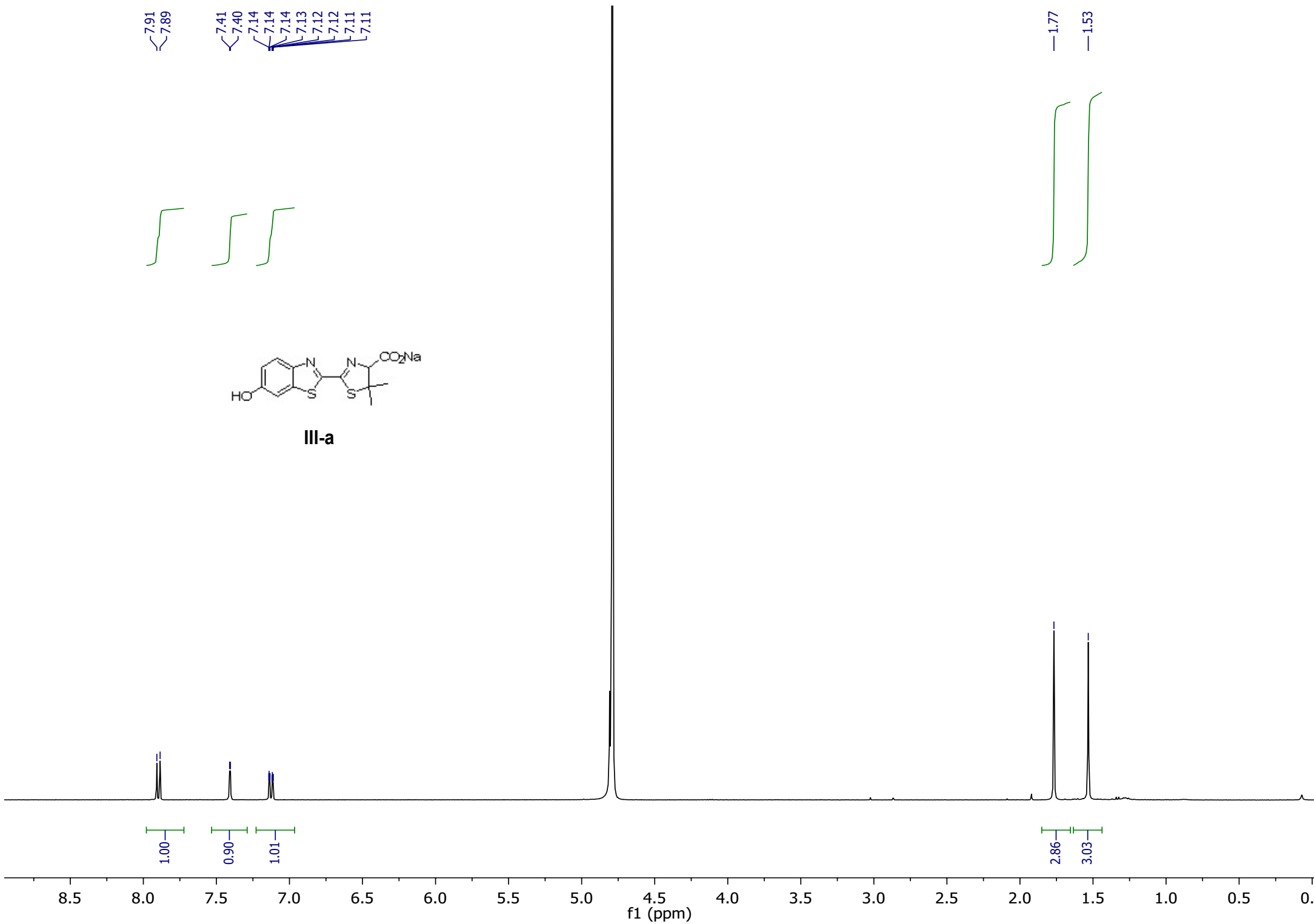

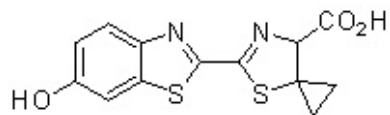

III-b

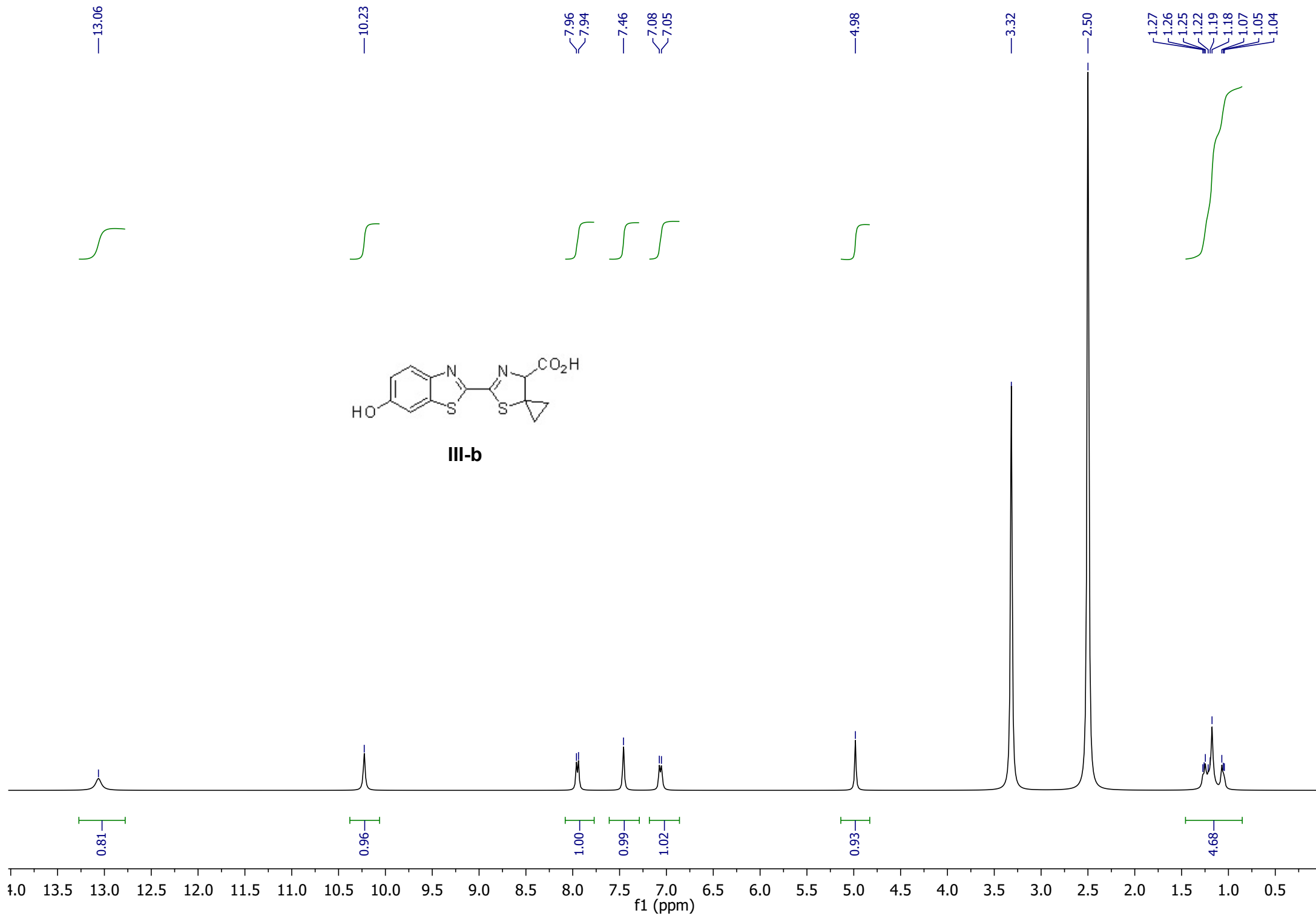

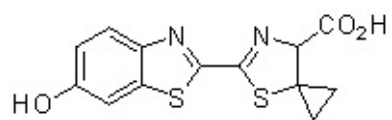

III-b

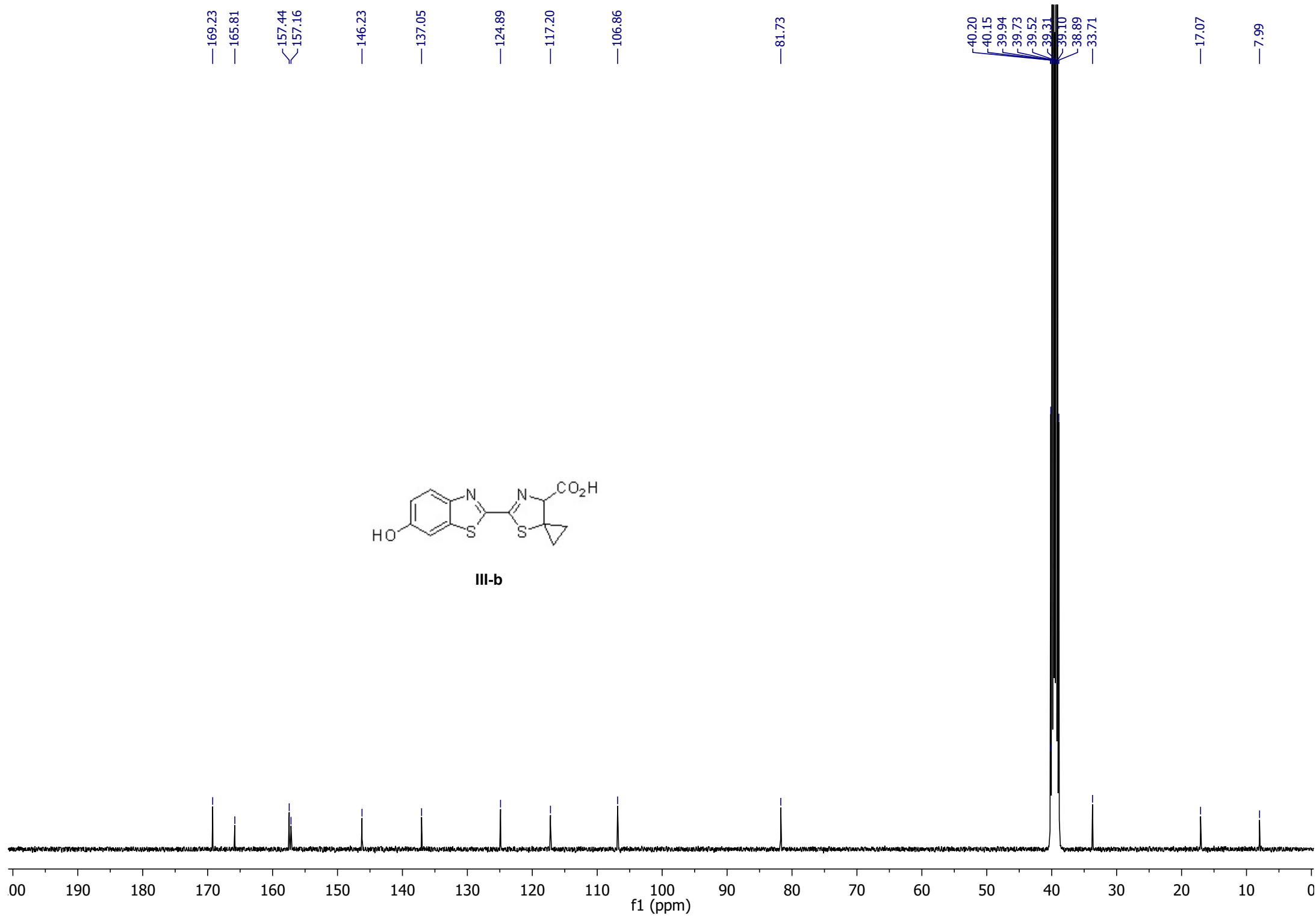

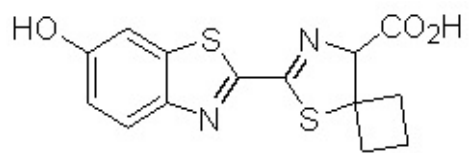

### III-C

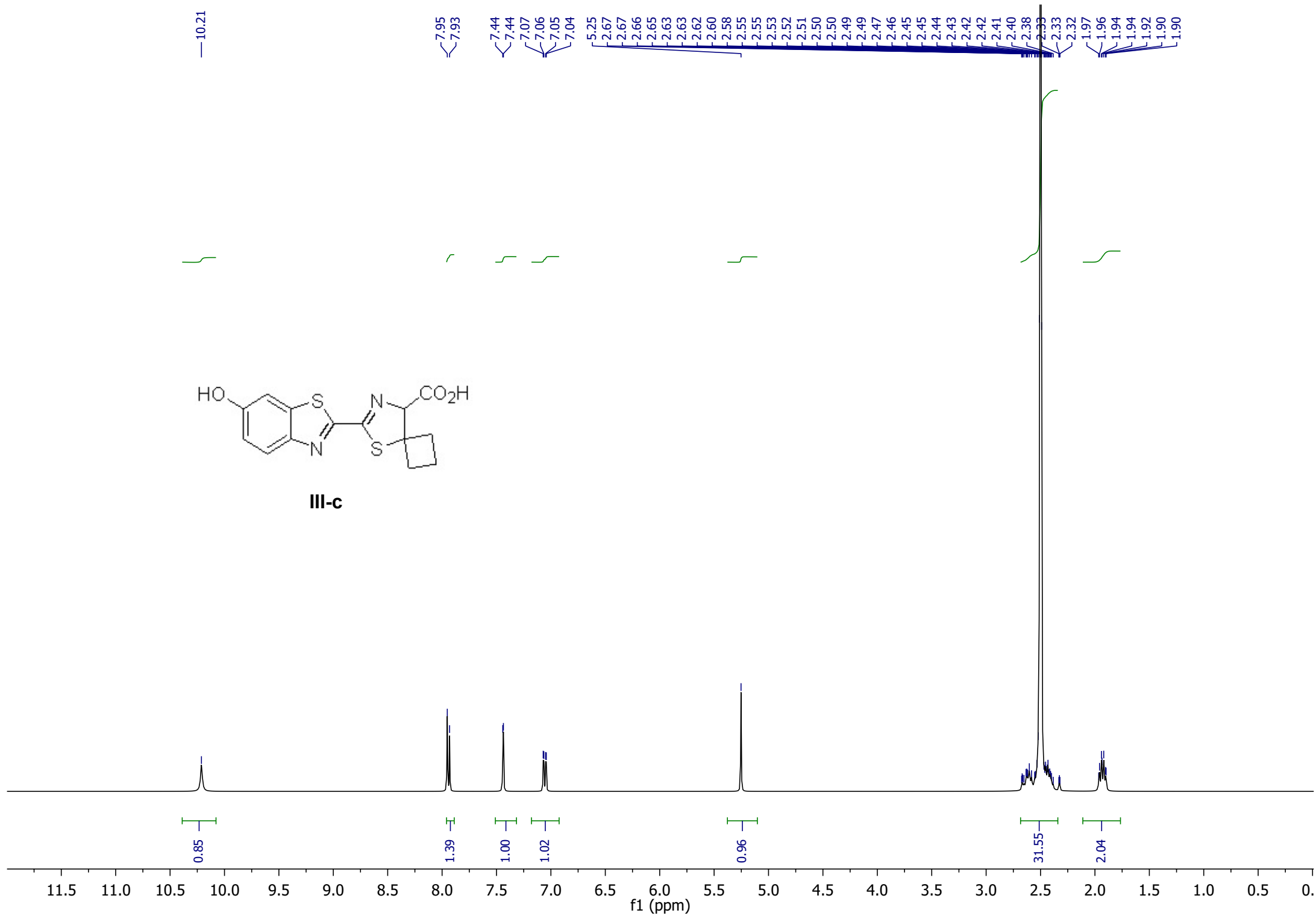

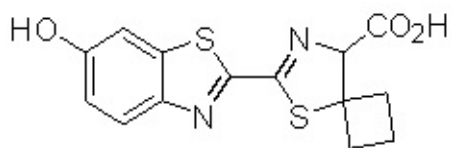

III-c

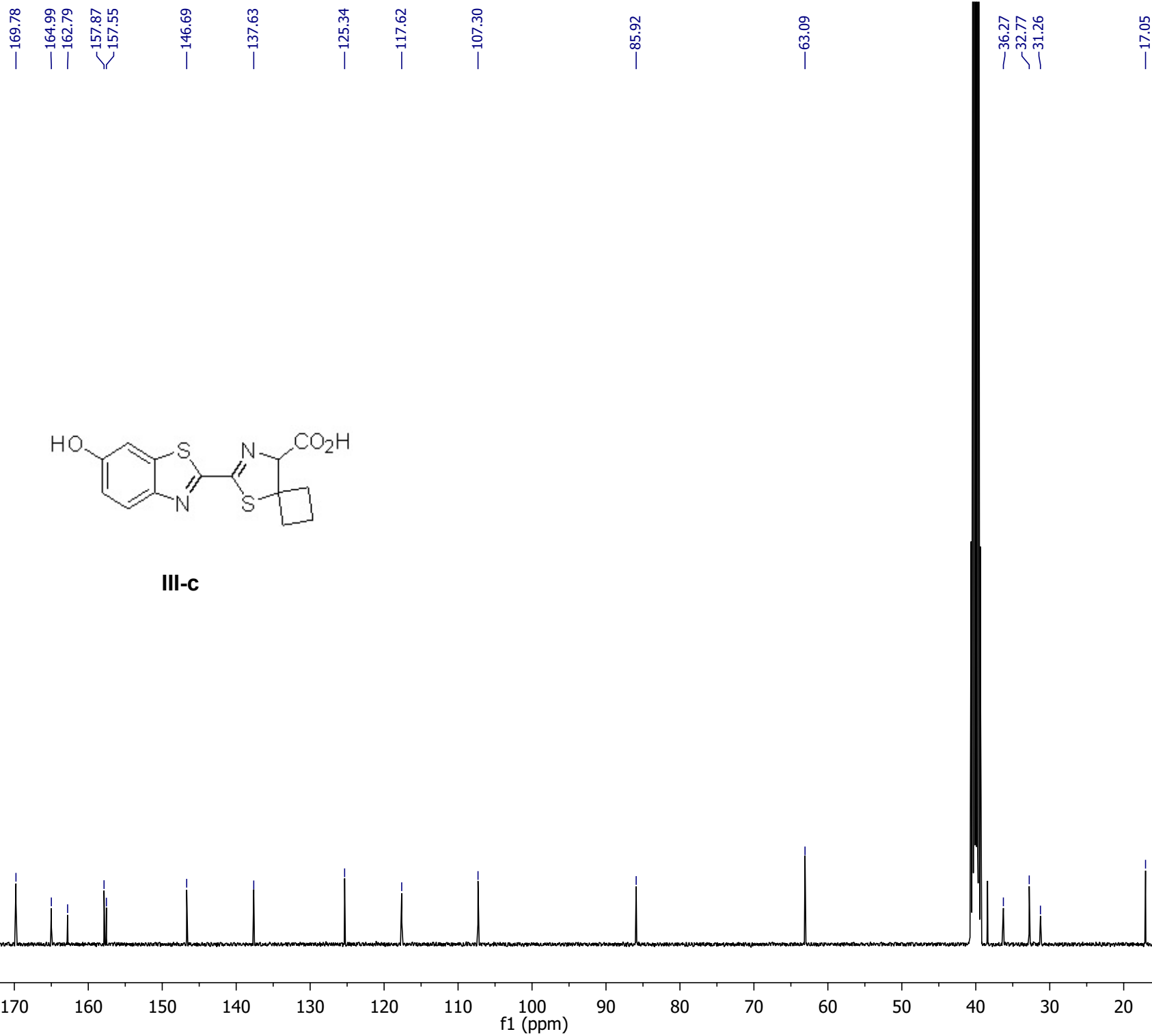

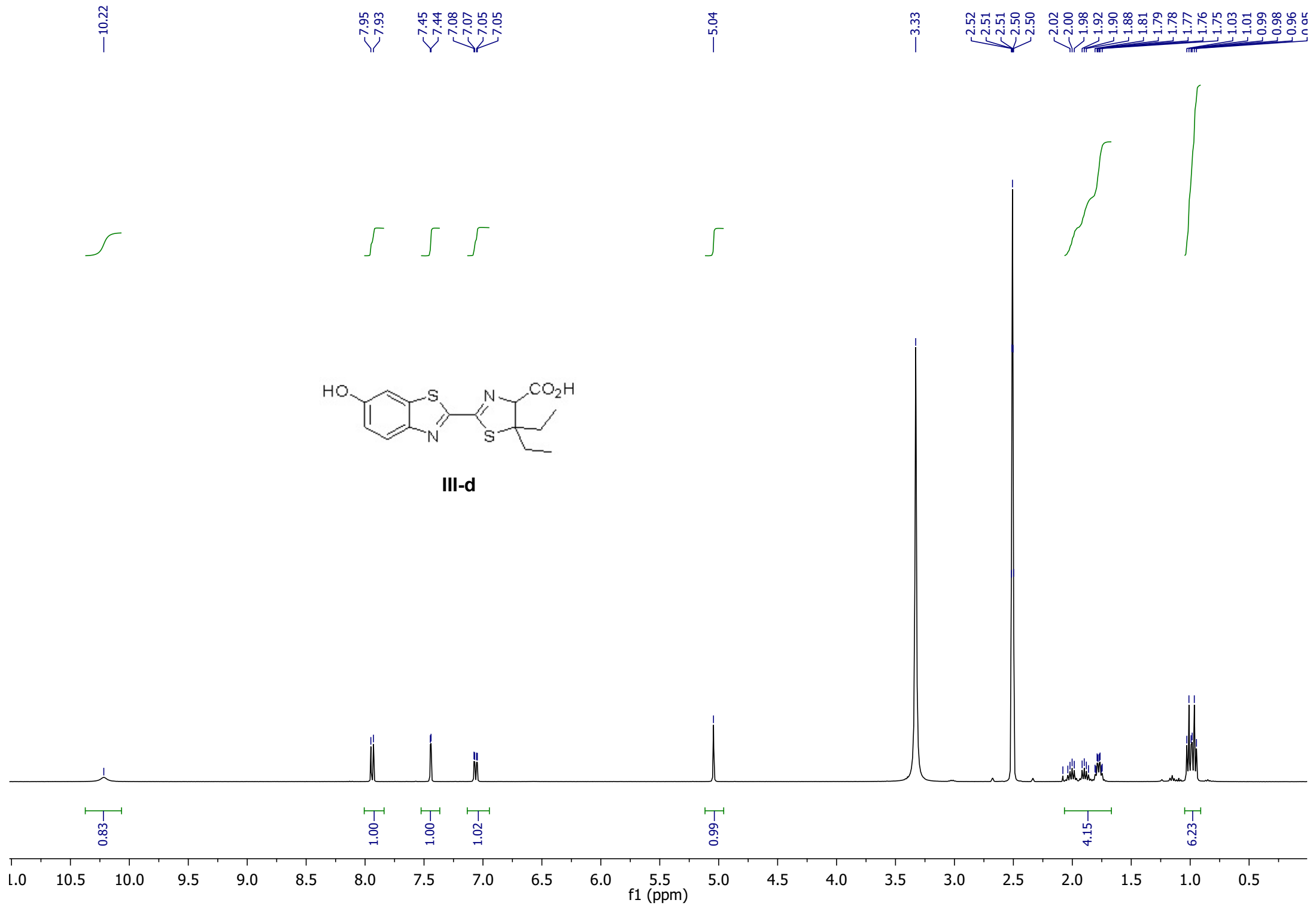

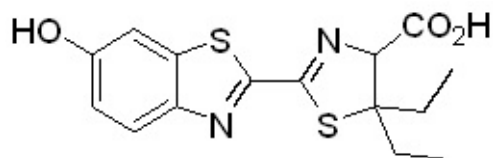

III-d

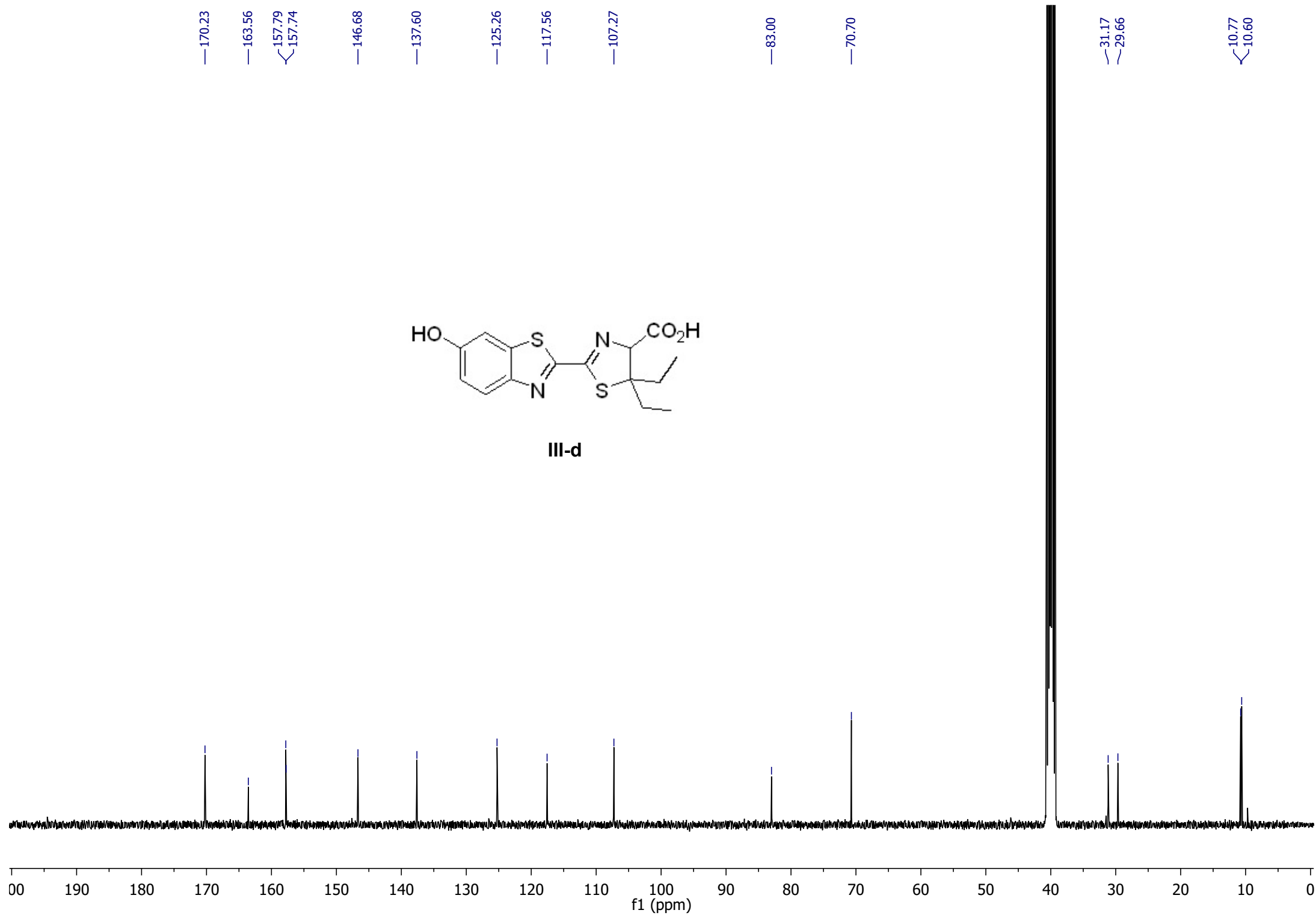

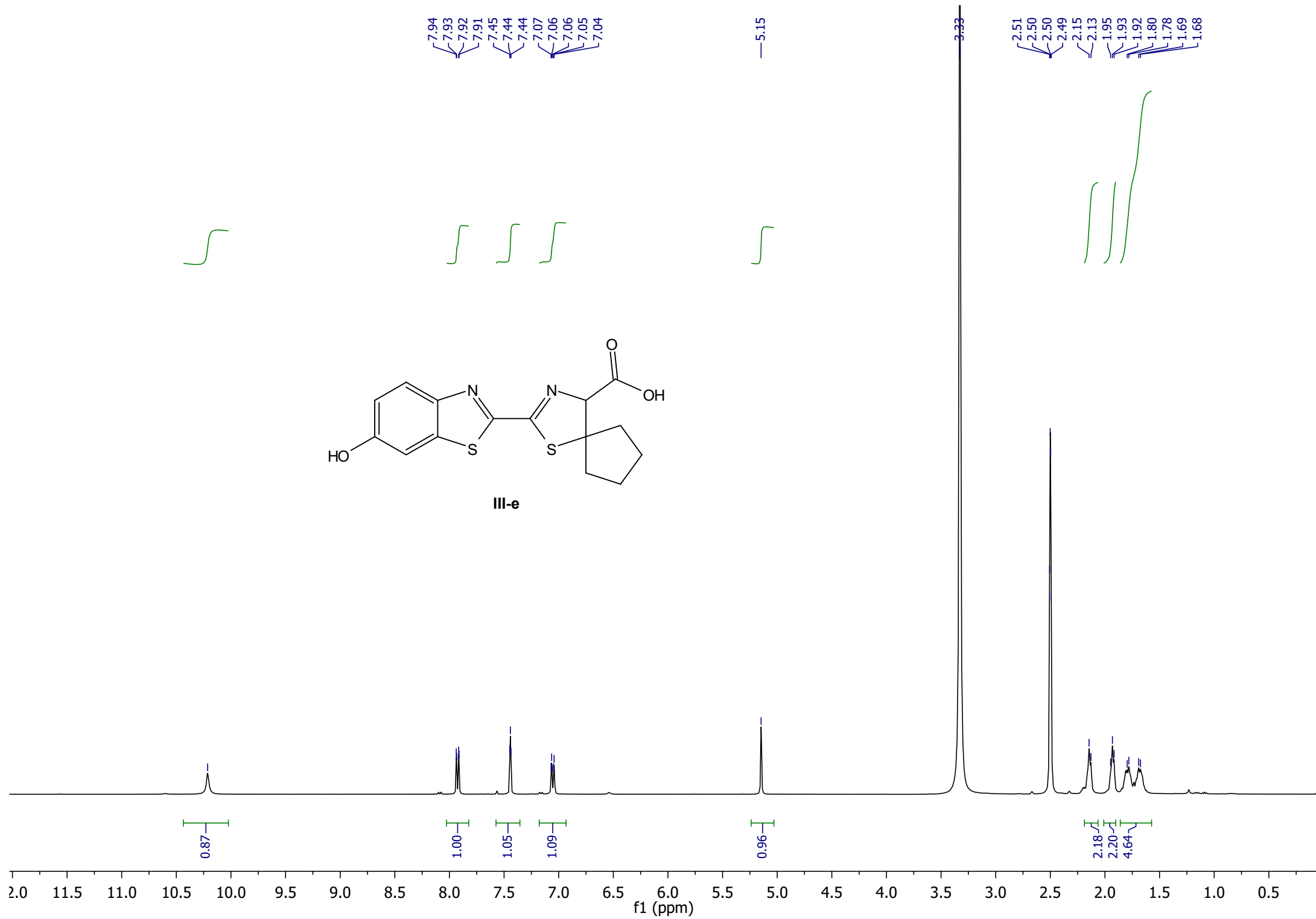

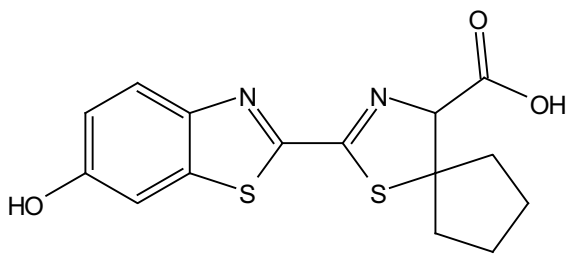

III-e

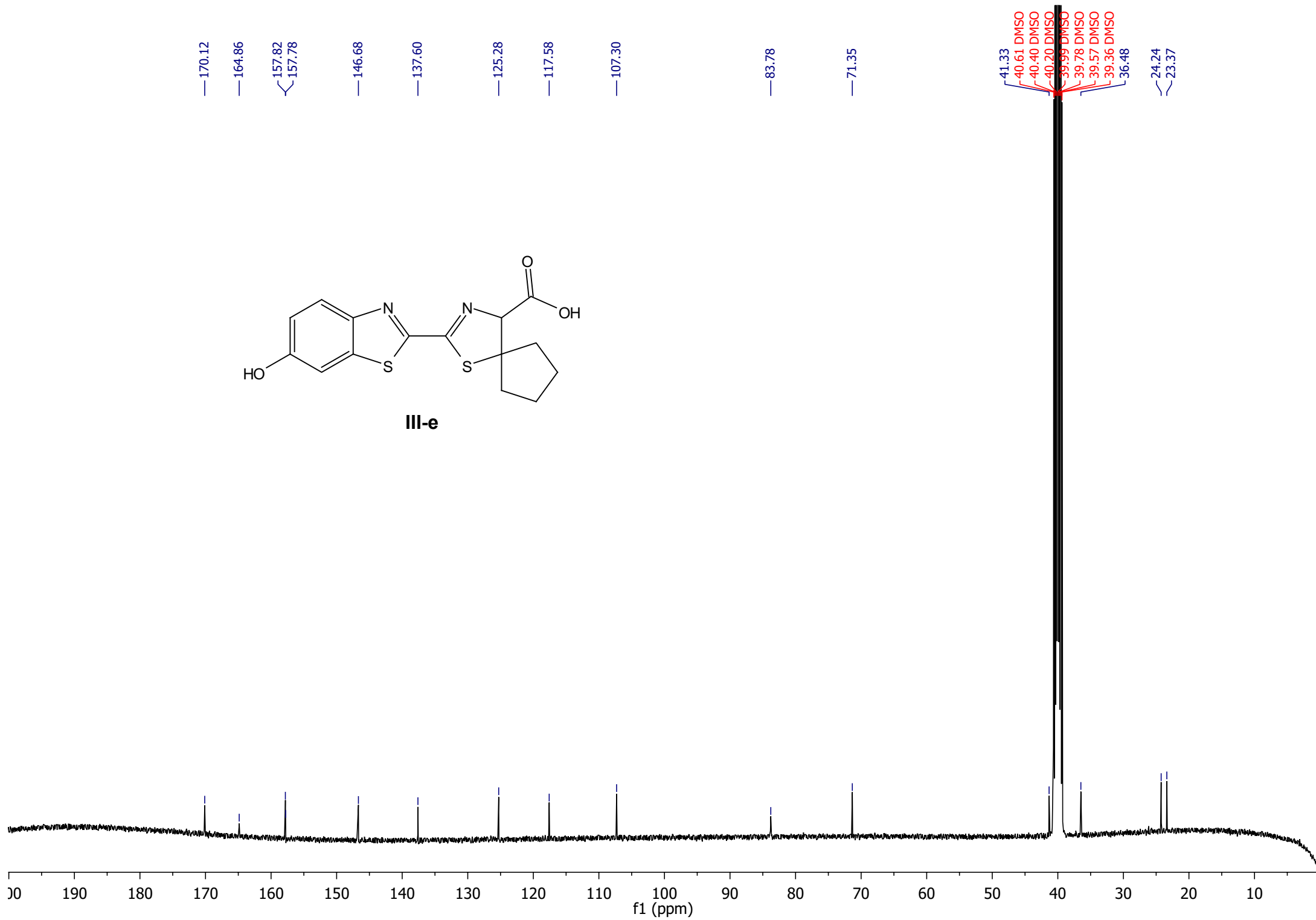

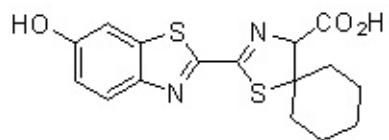

III-f

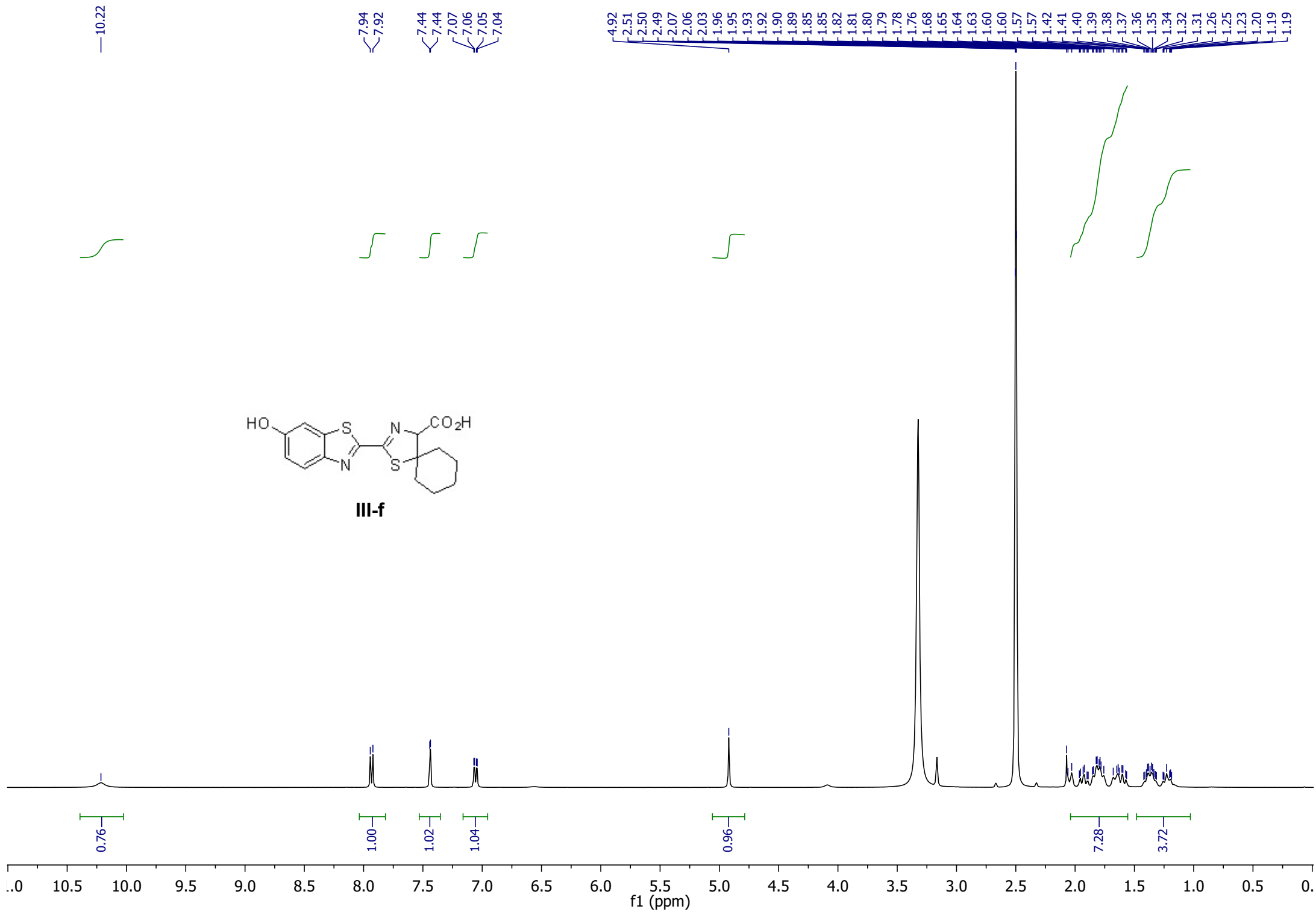

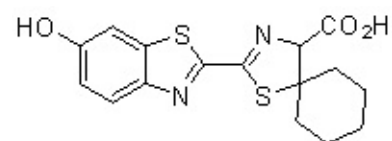

III-f

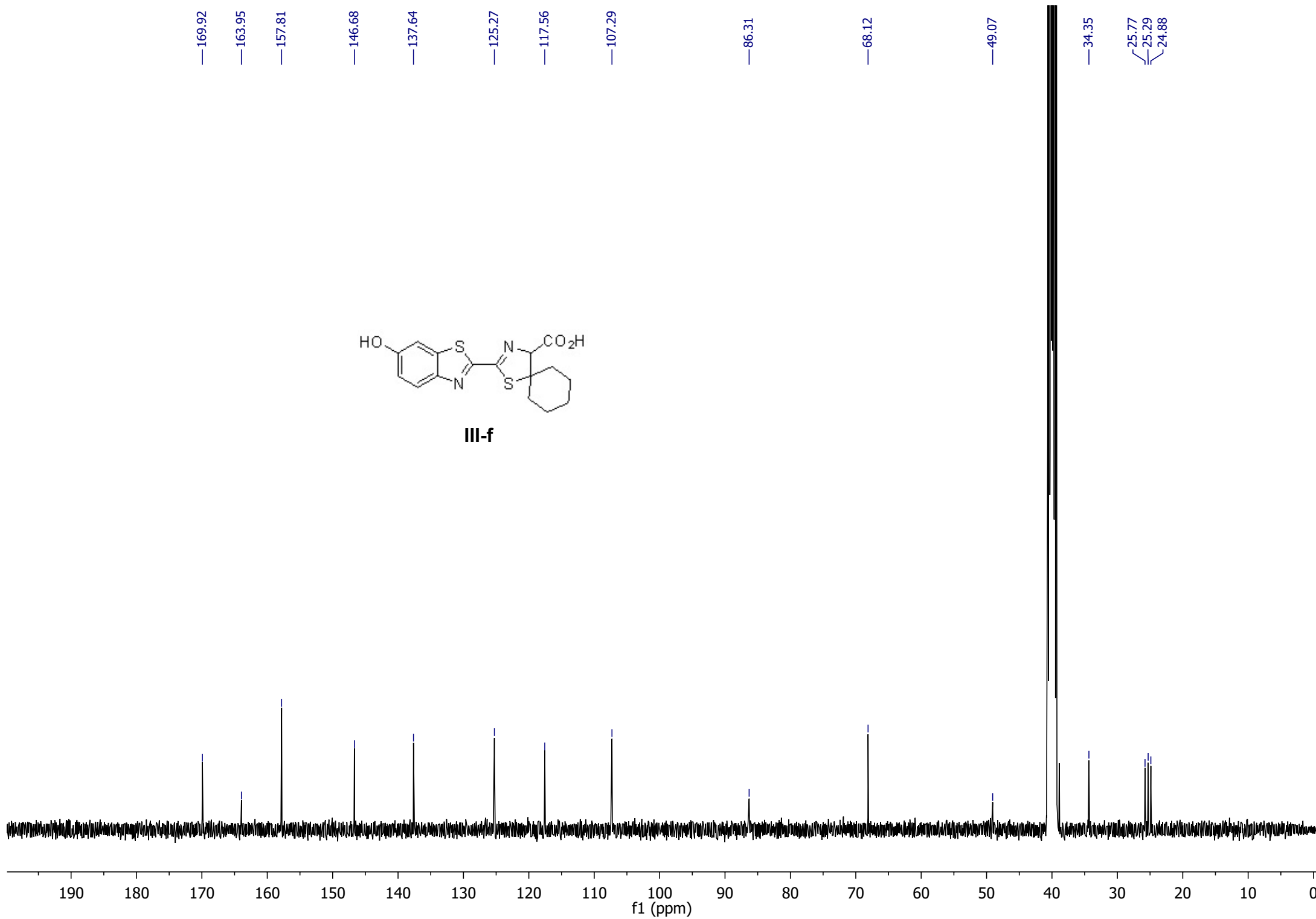

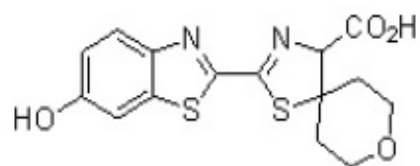

III-g

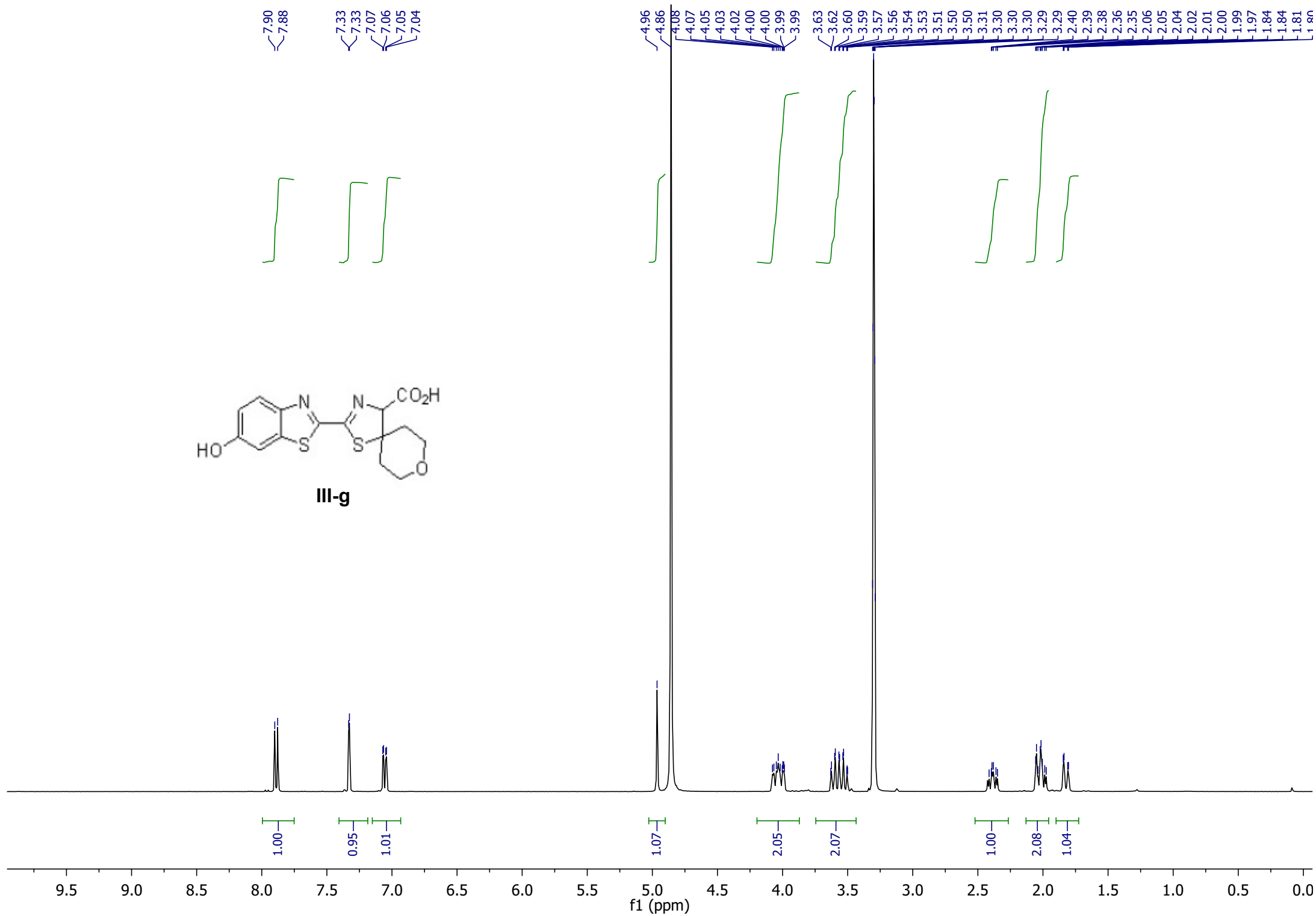

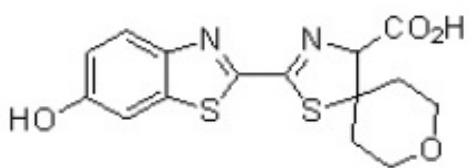

III-g

—171.37  
 —166.28  
 —159.07  
 —158.81  
 —148.12  
 —139.18  
 —125.93  
 —118.25  
 —107.36  
 —87.06  
 —68.35  
 —67.32  
 —65.82  
 —49.71  
 —49.64  
 —49.50  
 —49.43  
 —49.28  
 —49.21  
 —49.07  
 —49.00  
 —48.86  
 —48.79  
 —48.57  
 —48.36  
 —39.47  
 —35.89

200 190 180 170 160 150 140 130 120 110 100 90 80 70 60 50 40 30 20 10

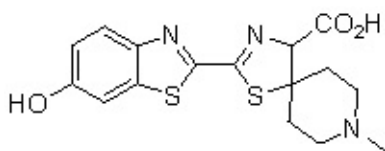

III-h

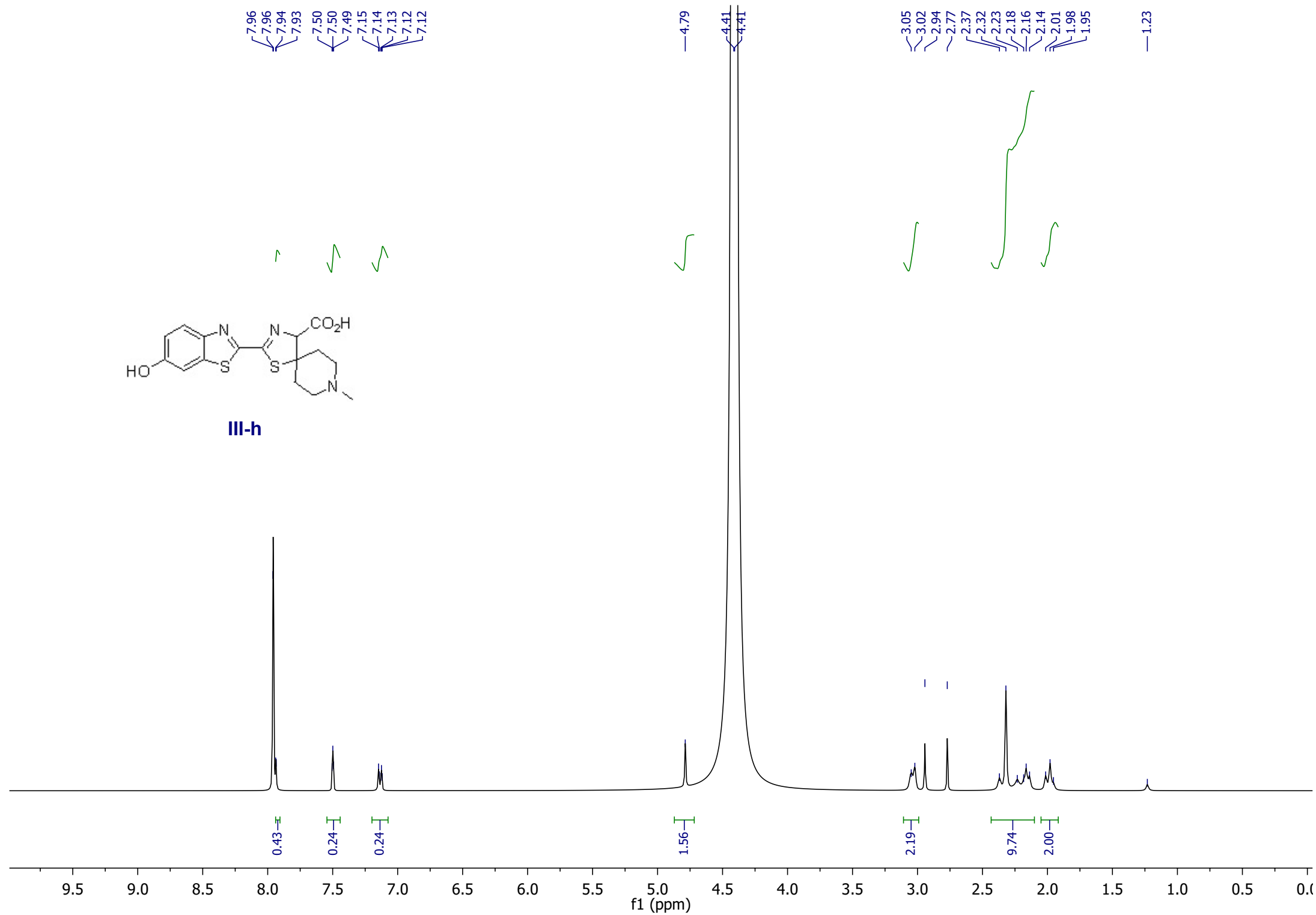

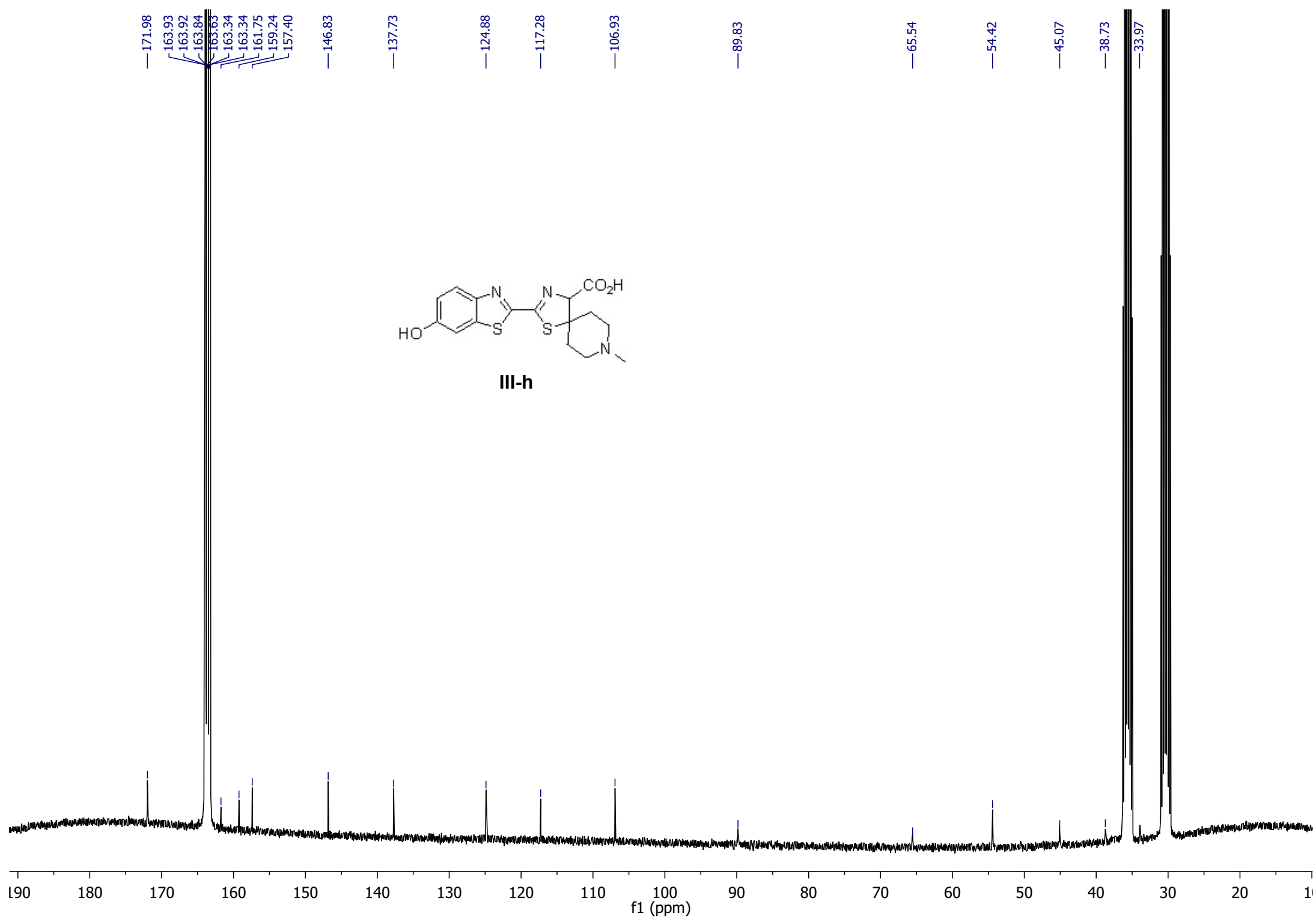

Supplement: S2 File — (PDF) [file pone.0243747.s002.pdf]
